# Structure-based identification of Jervine as a potent dual-targeting inhibitor of cell cycle kinases

**DOI:** 10.3389/fphar.2025.1662556

**Published:** 2025-11-21

**Authors:** Md Nayab Sulaimani, Khadija Imtiyaz, Md. Imtaiyaz Hassan, Fohad Mabood Husain, Aanchal Rathi, Mir Ovais Farooq, Anam Ashraf, Ravins Dohare, Sukhwinder Singh Sohal, Saba Noor

**Affiliations:** 1 Centre for Interdisciplinary Research in Basic Sciences, Jamia Millia Islamia, Jamia Nagar, New Delhi, India; 2 Department of Biosciences, Jamia Millia Islamia, New Delhi, India; 3 Department of Food Science and Nutrition, College of Food and Agriculture Sciences, King Saud University, Riyadh, Saudi Arabia; 4 Department of Biotechnology, Jamia Millia Islamia, New Delhi, India; 5 Department of Biotechnology, School of Life Sciences, Central University of Kashmir, Ganderbal, India; 6 Respiratory Translational Research Group, Department of Laboratory Medicine, School of Health Sciences, College of Health and Medicine, University of Tasmania, Launceston, TAS, Australia

**Keywords:** aurora kinase B, cyclin-dependent kinase 1, drug discovery, high-throughput screening, MD simulation, kinase inhibitors, natural products

## Abstract

Mitotic regulators play an essential role in cell cycle progression by ensuring correct chromosomal alignment, segregation, DNA replication, repair, and division, thereby maintaining genomic stability. Aberrant activity of cell cycle kinases, including aurora kinase B (AURKB) and cyclin-dependent kinase 1 (CDK1), might lead to disrupted mitotic checkpoints, causing aneuploidies and uncontrolled proliferation, which are critical hallmarks of cancers. Targeted inhibition of cell cycle kinases is an attractive strategy to combat cancers with minimal side effects. This study employed a comprehensive multi-staged computational approach to discover dual-targeting inhibitors against AURKB and CDK1, which are reported as key promoters of tumorigenesis. High-throughput screening of phytochemicals available in the Indian Medicinal Plants, Phytochemistry, and Therapeutics (IMPPAT) database was conducted to identify common lead/s from top hits. Jervine (IMPHY000366), a steroid alkaloid, emerged as a common compound depicting high binding affinity and ligand efficiency for AURKB and CDK1. In addition, this compound qualified all drug-like filters. After structure analysis, the docked complex was subjected to 300 ns MD simulation studies, confirming structural integrity in AURKB and CDK1 upon binding of Jervine. H-bonding pattern, secondary structural content, free energy landscape, and principal component analysis further supported Jervine’s strong and stable affinity for AURKB and CDK1. Lastly, MMPBSA showed a higher negative free energy in the presence of Jervine than VX-680 when complexed with AURKB. Finally, our results suggest that Jervine is a potent, dual-targeting kinase inhibitor with favourable pharmacokinetic and therapeutic properties, warranting further experimental validation for anticancer drug development.

## 1 Introduction

Aberrant mitotic regulation triggers defective chromosome segregation, chromothripsis, improper mitotic spindle assembly, and compromised genome stability (Potapova and Gorbsky, 2017). Moreover, dysregulated mitosis, causing variability in DNA, genetic alterations, and aneuploidies, drives tumor initiation and progression (Zhang et al., 2024). Consequently, mitotic regulators are recognised as critical therapeutic targets for treating various diseases, including cancer (Chan et al., 2012). Additionally, current cancer treatment modalities, including chemo-radiation and immunotherapy, are primarily associated with severe complications due to persistent damage to healthy cells, a lack of efficacy, and a targeted approach (Chan et al., 2012; Juthani et al., 2024). Hence, there is increasing interest among pharmaceutical industries and researchers in developing new therapeutics against oncogenic molecular targets. Selective targeting of mitotic mediators has been a long-standing anticancer strategy (Manchado et al., 2012); however, the challenges, including toxicity and drug resistance, favour failure to achieve complete tumour regression and elimination.

Chromosomal passenger complex (CPC) comprises crucial components, including aurora kinase B (AURKB), inner centromeric protein (INCEP), cyclin-dependent kinase 1 (CDK1) and survivin, playing an essential role in microtubule assembly and chromosomal segregation during cellular mitosis (Kitagawa and Lee, 2015). AURKB is a family of serine/threonine kinases involved in key mitotic processes that maintain genome stability and proper cell division (Bolanos-Garcia, 2005). Many such kinases are linked with human cancer initiation and progression. The recent discoveries of small-molecule kinase inhibitors for treating diverse types of cancer have also proven successful in clinical therapies (Bhullar et al., 2018; Shibuya et al., 1992). Although kinase-based drug targets are well explored, inhibition of distinct signalling pathways involving kinases is expected to be less cytotoxic to non-cancerous cells, with substantially lower toxic manifestations (Davies et al., 2000; Druker et al., 2006). Interestingly, specific-kinase inhibitors such as imatinib and dasatinib showed favorable outcomes compared to conventional anti-cancer therapies (Lombardo et al., 2004).

Overexpressed AURKB is common in pro-tumorigenic pathways involved in many cancer types, including lung, prostate, breast and liver (Borah and Reddy, 2021; Nakano et al., 2017). Drug resistance to anticancer therapy has also been associated with AURKB expression in several tumor types (Ahmed et al., 2021). Recent studies reveal that regulating AURKB and CPC localization activity is also a crucial surveillance mechanism for the orderly mitotic exit (Kitagawa and Lee, 2015). Several clinical inhibitors of AURKB, such as barasertib (AZD1152) (Wilkinson et al., 2007), have been evaluated for their ability to disrupt mitotic progression and induce apoptosis in cancer cells. However, challenges with toxicity and resistance have limited their clinical success. In physiological conditions, CDK1, a mitosis-promoting factor, is also a vital component of CPC and, in complex with cyclin-B1, induces G2/M and G1/S transitions along with G1 progression (Kalous et al., 2020). CDK1 is reported to exhibit high oncogenic potential when aberrantly expressed and is associated with several cancers, including melanoma and lung cancer (Wang et al., 2023). Several small molecules targeting CDK1 or multiple CDKs have been developed and are under pre-clinical evaluation (Flynn et al., 2015; Zeng et al., 2008).

Ample evidence has suggested that the mitigation of mitotic regulators such as AURKB and CDK1, when aberrantly expressed, can prevent compromised genomic integrity of cells showing abnormal mitotic regulation (Akl et al., 2022; Kang et al., 2014; Löwenberg et al., 2011; Soncini et al., 2006). Concomitant inhibition of multiple cancer-driving kinases is an established and effective strategy to improve clinical responses to conventional targeted therapies (Ciceri et al., 2014). Since AURKB and CDK1 are crucial components of CPC, their cumulative inhibition could be an attractive strategy for drug development to combat associated diseases. This study aimed to target AURKB and CDK1, regulators of CPC assembly, by identifying novel and effective dual-functioning small-molecule compounds. Till now, several small-molecule inhibitors have been developed against AURKB or CDK1 individually, but highly efficacious and specific inhibitors that could simultaneously target both enzymes and render CPC inactivation are still scant.

Recently, the drug development strategy has mainly focused on inhibitors encroaching and occupying ATP-binding pockets of kinases (Nakano et al., 2017; Umezawa and Kii 2021). This study aimed to logically identify specific and effective molecules that could selectively recognise the catalytic region of AURKB and CDK1 and exhibit strong affinity and selectivity. *In-silico* tools have played an essential role in designing and developing small-molecule inhibitors based on structure-activity relationships. Pharmacophore remodelling, molecular docking, and 3D-QSAR helped identify key structural features of ligands for targeted AURKB inhibition (Ashraf et al., 2021; Selvam et al., 2025). Similarly, advanced computational analysis involving scaffold repurposing and QSAR has documented inhibitors against CDK1 (Al-Sha’er and Taha, 2010; Elkamhawy et al., 2021).

A multitier virtual screening approach was applied systematically to identify dual-specific molecules that could particularly target AURKB and CDK1 for potential therapeutic applications. The commercially available Indian Medicinal Plants, Phytochemistry and Therapeutics (IMPPAT) database, comprising approximately ⁓18,000 natural compounds, was virtually screened to identify common lead/s. Furthermore, a sequential filtering process using Lipinski’s rule of 5, the PAINS filter, ADMET properties, and prediction of activity spectra of substances (PASS) analysis was employed to identify a familiar and promising candidate against AURKB and CDK1. Subsequently, an all-atom molecular dynamics (MD) simulation was conducted on detected potential hits (Naqvi et al., 2018).

The findings of the multistage filtering strategy revealed a familiar candidate IMPHY000366, also known as Jervine, against AURKB and CDK1. Jervine is a steroidal alkaloid known for its anti-neoplastic characteristics. It interferes with cellular mitosis through selective targeting of hedgehog and AKT signalling pathways. The pharmacokinetic profiling, ADMET analysis, and PASS evaluation project Jervine with strong drug-likeliness. An extensive computational analysis and MD simulation study depicted strong binding affinities, interaction dynamics with key residues of both enzymes, and enhanced ligand efficiencies.

The binding dynamics and potential of Jervine were evaluated in comparison to the established inhibitors of AURKB and CDK1, which are VX-680 and RO-3306, respectively, and Jervine demonstrated superior binding affinities and ligand efficiencies relative to these control drugs. MMPBSA analysis was also conducted to validate the results of virtual screening and docking. The identified hit Jervine showed higher binding affinities. This dual-targeted approach aligns with emerging strategies in cancer therapeutics aimed at co-inhibiting key cell cycle regulators to enhance effectiveness and reduce drug resistance.

## 2 Materials and methods

### 2.1 Structure retrieval and refinement

The manually curated phytochemical library of medicinal plants, Indian Medicinal Plants, Phytochemistry and Therapeutics (IMPPAT) 2.0, constitutes approximately ⁓18,000 compounds. A total of 11,699 compounds remained after applying Lipinski’s Rule of Five (RO5), which were subsequently used for virtual screening. The RCSB Protein Data Bank was used to obtain 3D structures of AURKB (PDBID: 4AF3) and CDK1(PDBID: 6GU2), followed by pre-processing that includes elimination of all the heteroatoms by using PyMod 3 (an open-source plugin of PyMol) (Janson and Paiardini, 2021). The co-crystallised ligand and water molecules were removed from the structure to conduct virtual screening. This was done to ensure that the associated ligand and water molecules do not influence the binding patterns and affinities of drugs being screened. The protein underwent import, refinement, review, modification, and reduction during preparation. Using the Prime tool, the protein production wizard filled in the lack of side chain residues. The catalytically essential residues and active sites were retained in the protein structure. Utilising a CHARMM force field, energy reduction was employed to create low-energy state proteins, which were then utilised for molecular modelling. Simultaneously, the PDBQT files of compounds from the IMPPAT library were downloaded.

### 2.2 Molecular docking analysis

To determine binding affinities and mechanisms, molecular docking was conducted with InstaDock software with a blind search space against the IMMPAT library compounds (Mohammad et al., 2021). For the molecular docking of AURKB protein and phytochemicals, the center coordinates was at X: 16.886, Y: −16.521, Z: 0.544, and dimensions of X: 58, Y: 71, Z: 75, with a grid spacing of 1 Å and for CDK1, the search box was defined by setting a grid with the center coordinates at X: 315.904, Y: 216.746, Z: 191.792, and dimensions of X: 76, Y: 60, Z: 58, with a grid spacing of 1 Å. The top hits were obtained based on affinity score, and docked conformers were created for interaction studies. The binding affinities between the ligand and protein were calculated using the QuickVina-W (Mohammad et al., 2021). PyMol viewer and BIOVIA/Discovery Studio 2017R2 platforms were employed for computational analysis and structure visualization of docked structures.

Additionally, to validate the AURKB and CDK1 targeting potential, the docking profile of selected hit/s was compared with positive controls of both enzymes. To get deeper insights, close interactions were viewed in Pymol software and polar contacts within the range of 3.5 Å were measured. Common compound/s from the top hits depicting interactions with ATP-binding pockets of both enzymes were selected for further evaluation. In addition, molecular docking studies were also performed using AURKB and CDK1 with their respective known inhibitors, VX-680 and RO-3306, which served as controls.

### 2.3 Physicochemical properties

The common compound selected after molecular-based drug screening was checked for physicochemical properties using SwissADME tools. The physicochemical properties, drug likeliness, lipophilicity, Lipinski rule, and other parameters were noted. The Pan Assay Interference Compounds (PAINS) filter was implemented to ensure specificity in drug design and discovery. This filter helps to eliminate compounds displaying structural patterns that tend to bind multiple targets in a non-specific way. Compounds with high PAINS value are notorious and interfere with biological assays through non-specific binding and are therefore not considered good drug candidates. In addition to Jervine, a similar analysis was also done for VX-680 and RO-3306 drugs as controls.

### 2.4 ADMET analysis

Jervine’s pharmacokinetics and physicochemical features were checked to evaluate efficacy using pkCSM (Pires et al., 2015) and SwissADME tools (Daina et al., 2017). ADMET analysis is crucial in drug discovery as it reduces the risk of drug failure in clinical trials. Jervine, VX-680, and RO-3306 were probed for ADMET properties using the SMILES format file for pkCSM generated using the Discovery Studio software (Alshehri et al., 2024).

### 2.5 PASS evaluation

PASS analysis was performed to critically evaluate the biological and pharmacological features of Jervine, VX-680 and RO-3306 after evaluating ADMET features (Lagunin et al., 2000). Based on a pre-defined structure-activity relationship, the PASS web server provides a comprehensive understanding of the pharmacological effects of drug molecules. The analysis results are presented as P*a* (probability of activity) and P*i* (probability of inactivity) and generally compounds with a high P*a*/P*i* ratio are considered to have biological potential.

### 2.6 Interaction studies

After a thorough exploration of the pharmacological role of Jervine, the interaction analysis between Jervine with AURKB and CDK1 was performed. 2D interaction analysis was conducted with Discovery Studio software, and critical residues of the ATP binding pocket of enzymes making hydrogen bonds, hydrophobic interactions, and other interactions with the Jervine were identified. The formation of multiple noncovalent interactions, including hydrogen bonds, hydrophobic interactions, and π-π stacking, was noted between Jervine and the key residues of AURKB and CDK1. For comparison, VX-680 and RO-3306 were also checked for interactions with AURKB and CDK1, respectively. In PyMol, the hydrogen bonds at 3.5Å were identified and highlighted/marked with dotted lines inside the protein-ligand complex.

### 2.7 MD simulation studies

Molecular dynamics (MD) simulation is a computer method used to investigate the molecular structure and changes in the conformation of a protein caused by the binding of a ligand (Hassan et al., 2022; Khan et al., 2021). The MD simulation experiments confirmed the docking results of AURKB and CDK1 with Jervine as a standard drug. VX-680 and RO-3306 (CDK1 inhibitor) were taken as a positive control. GROMACS v5.5.1 with the CHARMM force field was used to mimic the structural features of AURKB and CDK1 and their docked complexes with Jervine and positive controls. This is an open-source software program that is mainly used to simulate the behaviour of biomolecules and assist in the process of designing drugs using computers. The topologies of the receptor-ligand complex were generated using a web-based CgenFF (https://cgenff.com/) server. The three systems for each enzyme were submerged in a cubic container using the TIP3P water model for solvation.

To ensure charge neutrality, the simulated systems were neutralized by adding the requisite counter-ions (Na^+^ and Cl^−^). Specifically, six Cl^−^ ions were introduced into the AURKB systems and four Cl^−^ ions into the CDK1 systems, achieving electrostatic balance before solvation and energy minimization. The solvated complexes were energy-reduced using the conjugate gradient technique in conjunction with 1,500 steps of the steepest descent approach to eliminate any potential steric hindrances between the atoms. Under constant volume circumstances, the equilibration procedure was carried out in two phases throughout 1,000 ps. The temperature was gradually increased from 0 to 300 K while maintaining a pressure of 1 atm. An in-depth examination of the MD data was conducted using GROMACS integrated capabilities. A 300 ns MD simulation was conducted. Subsequently, the QtGrace program was used to analyse the resulting trajectories.

### 2.8 MMPBSA analysis

MMPBSA (Molecular mechanics/Poisson-Boltzmann surface area) is one of the most widely used approaches for estimating the binding free energy of a protein-ligand complex (Genheden and Ryde, 2015). A short MD trajectory of 10 ns (from 290 ns to 300 ns) was extracted from the stable region of the AKB-Jervine and AURKB-VX-680 complexes for MMPBSA calculations. A similar experiment was conducted for CDK1-Jervine and CDK1-RO-3306 (control). The binding energy components were calculated using the MMPBSA approach of the gmx_mmpbsa package. The gmx_mmpbsa tool uses the following equation to calculate the binding energy of the protein-ligand complex-

$$\Delta G_{\text{Binding}}=G_{\text{Complex}}-\left(G_{\text{Protein}}+G_{\text{Ligand}}\right)$$

where *G*_Complex_ signifies the total free energy of the binding complex, and *G*_Protein_ and *G*_Ligand_ are the measures of total free energies of native protein and the compounds Jervine, VX-680, and RO-3306.

## 3 Results

### 3.1 Molecular docking-based virtual screening

Computational methods are exploited to virtually screen and identify potential molecules against pre-defined therapeutic targets. The implication of high-throughput screening methodologies also reduces experimental errors and time consumption for lead identification. Molecular docking-based virtual screening was conducted using InstaDock software to discover high-affinity binding partners of AURKB and CDK1. The top 50 hits were initially selected based on binding affinity scores, and common compounds for AURKB and CDK1 were identified. In the findings, IMPHY000366 (Jervine) was identified as a common compound against AURKB and CDK1 with binding affinities of −10.7 and −9.1 kcal/mol, respectively. The binding affinities of AURKB-Jervine and CDK1-RO-3306 were found to be −8.3 and −9.2 kcal/mol (Supplementary Tables S1 and S2). Comparing it to other clinically approved inhibitors, Jervine demonstrates promising potential as a dual AURKB/CDK1 inhibitor. For AURKB, barasertib (AZD1152-HQPA), a selective AURKB inhibitor in clinical development, exhibits sub-nanomolar potency (IC_50_ ≈ 0.37 nM). Jervine’s predicted binding affinity of −9.1 kcal mol^−1^ (∼250 nM Kd) falls within the high-nanomolar range, indicating favorable binding and suggesting its relevance as a scaffold for further optimization. For CDK1, Jervine showed a binding affinity of −10.7 kcal mol^−1^ (∼16 nM Kd), comparable to clinically studied multi-CDK inhibitors. Notably, AT7519 inhibits CDK1 with IC_50_ ≈ 210 nM and Ki ≈ 38 nM, meaning Jervine demonstrates markedly stronger predicted binding. Together, these comparisons emphasize Jervine’s potential as a competitive inhibitor for both AURKB and CDK1 (Squires et al., 2009). To assess the robustness of our docking protocol, we performed multiple independent docking runs of Jervine with CDK1 and AURKB using different random seed values. Interestingly, all runs consistently yielded the same docking score and binding pose, indicating that the results are highly reproducible and independent of the initial search conditions. This convergence strongly supports the reliability of the predicted binding mode. These data are provided in the Supplementary Material (Supplementary Tables S3 and S4).

### 3.2 Physicochemical properties

Physicochemical profiling of a drug molecule is a crucial step in rational drug design, as it allows for the analysis and optimisation of pharmacological efficacy and therapeutic application. Physicochemical properties and drug likeliness of Jervine were evaluated using SwissADME software and are provided in Supplementary Table S5. Characteristics, including molecular weight, Lipinski rule, TPSA, and lipophilicity, provided more profound insights into the drug-like behaviour of Jervine.

### 3.3 ADMET analysis

Among the top 50 hits from the docking study, Jervine was identified as a common molecule with high binding affinities towards AURKB and CDK1. PkCSM and SwissADME web servers were used to predict the ADMET properties of Jervine. ADMET prediction encompasses a set of different parameters depicting pharmacokinetic properties and threshold values that help eliminate unsuitable drugs. ADMET analysis was conducted for Jervine and the positive controls of AURKB and CDK1, VX-680 and RO-3306, respectively. It was found that Jervine had a higher gastrointestinal (GI) absorption (94.78%) compared to VX-680 (75.98%) and RO-3306 (92.07%). Jervine was surprisingly identified as a substrate of the organic cation transporter 2 (OCT2), whereas VX-680 and RO-3306 did not exhibit substrate characteristics for this transporter. Compared to VX-680 and RO-3306, Jervine demonstrated higher blood-brain barrier (BBB) permeability, as indicated by its elevated logBB value. Like VX-680, Jervine did not exhibit AMES toxicity, indicating an absence of mutagenic potential.

In contrast, RO-3306 tested positive in the AMES assay, suggesting potential genotoxicity. Table 1 provides the computed ADMET properties of Jervine, VX-680, and RO-3306. Jervine showed satisfactory ADMET properties, including high GI absorption, significant BBB permeation, low renal/hepatic toxicity, low AMES toxicity, inhibition of CYP2D, and the substrate of OCT2.

**TABLE 1:** ADMET propertie.

S. No.	Compound ID	Absorption	Distribution	Metabolism	Excretion	Toxicity
		GI Absorption	BBB permeation	CYP2D6 Inhibitor	OCT2 substrate	AMES
1.	Jervine	94.785	0.202	No	Yes	No
2.	VX-680	75.989	−1.346	No	No	No
3.	RO-3306	92.071	0.114	No	No	Yes

### 3.4 PASS evaluation

To learn about the pharmacological activity of Jervine in comparison to VX-680 and RO-3306, the PASS analysis was performed. The PASS tool has a comprehensive database of bioactive molecules and extensive information on structure-activity relationships from clinical and preclinical studies. Based on the values of P*a* (probable actives) and P*i* (probable inactives), the required biological property for a molecule can be elucidated. After the PASS analysis, it was found that all three compounds had anti-neoplastic properties with P*a*>0.7. Also, P*a* of Jervine (0.722) was higher than R0-3306 (0.442), but lower than VX-680 (0.893) (Table 2).

**TABLE 2:** Prediction of activity spectra of substance analysis.

S. No.	Compound ID	Pa	Pi	Biological activity
1.	Jervine	0.722	0.022	Antineoplastic
		0.652	0.001	Antineoplastic (bone cancer)
		0.464	0.016	Antineoplastic (lung cancer)
		0.446	0.009	Antineoplastic (ovarian cancer)
		0.428	0.009	Antineoplastic (non-small cell lung cancer)
1.	VX-680	0.893	0.002	Aurora kinase inhibitor
		0.887	0.005	Protein kinase inhibitor
		0.878	0.003	Protein-serine-threonine kinase inhibitor
		0.876	0.002	Aurora-B kinase inhibitor
		0.666	0.032	Antineoplastic
6.	RO-3306	0.442	0.023	Antineoplastic (solid tumors)
		0.405	0.026	Focal adhesion kinase 2 inhibitor
		0.325	0.003	Pim-2 kinase inhibitor
		0.322	0.004	Pim kinase inhibitor
		0.321	0.047	Focal adhesion kinase inhibitor

### 3.5 Interaction studies

**FIGURE 1:**
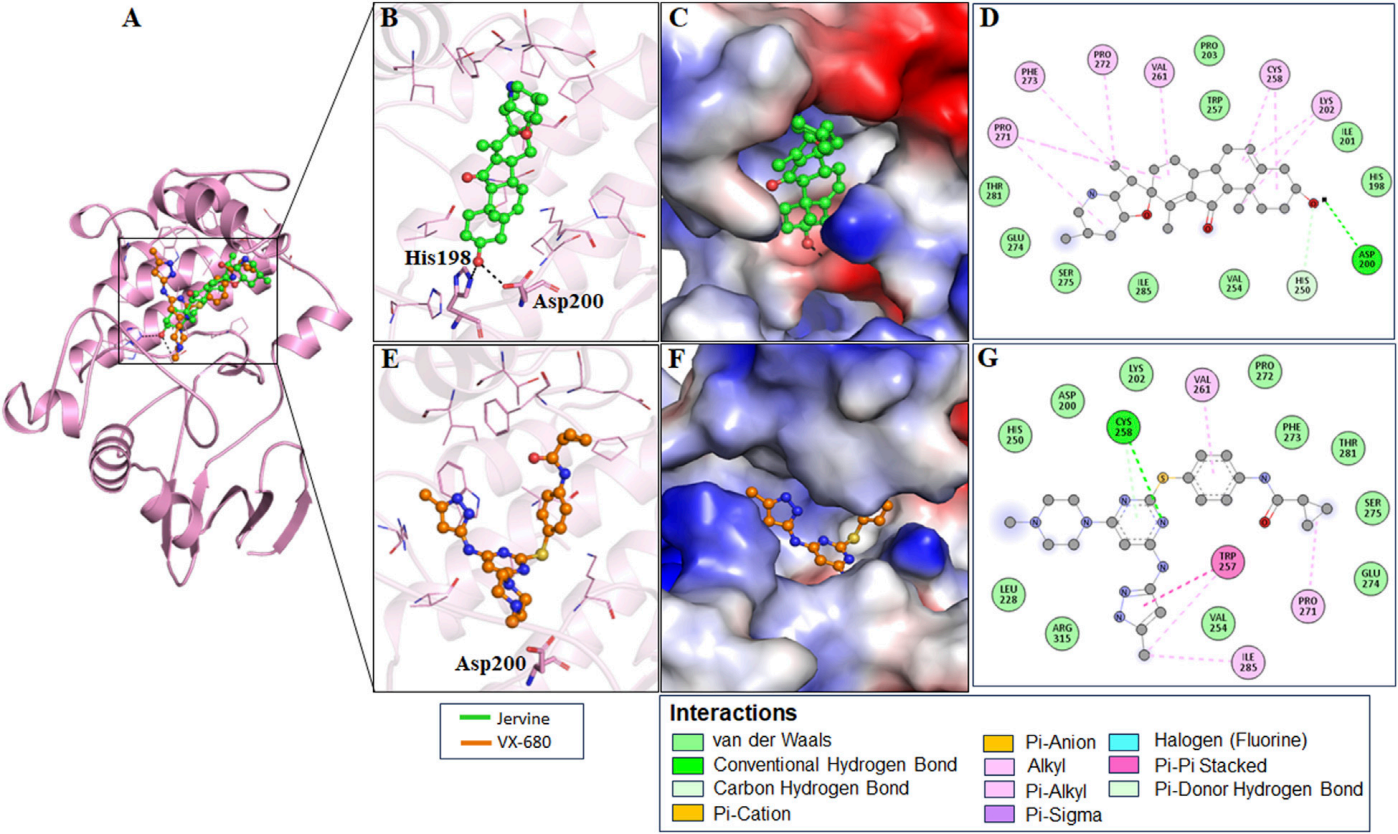
Structural representation of AURKB with ligands. Interaction analysis of AURKB with Jervine and VX-680. **(A)** 3D interaction of AURKB with Jervine and VX-680. **(B)** Enlarged view depicting the key amino acid residues involved in the interaction between AURKB and Jervine. **(C)** Surface electrostatic potential view of AURKB with Jervine. **(D)** 2D interaction diagram showing hydrogen bonds and hydrophobic interactions between AURKB and Jervine. **(E)** Enlarged view depicting the key amino acid residues involved in the interaction between AURKB and VX-680. **(F)** Surface electrostatic potential map illustrating the binding interface of AURKB with VX-680. **(G)** 2D interaction diagram showing hydrogen bonds and hydrophobic interactions between AURKB and VX-680.

**FIGURE 2:**
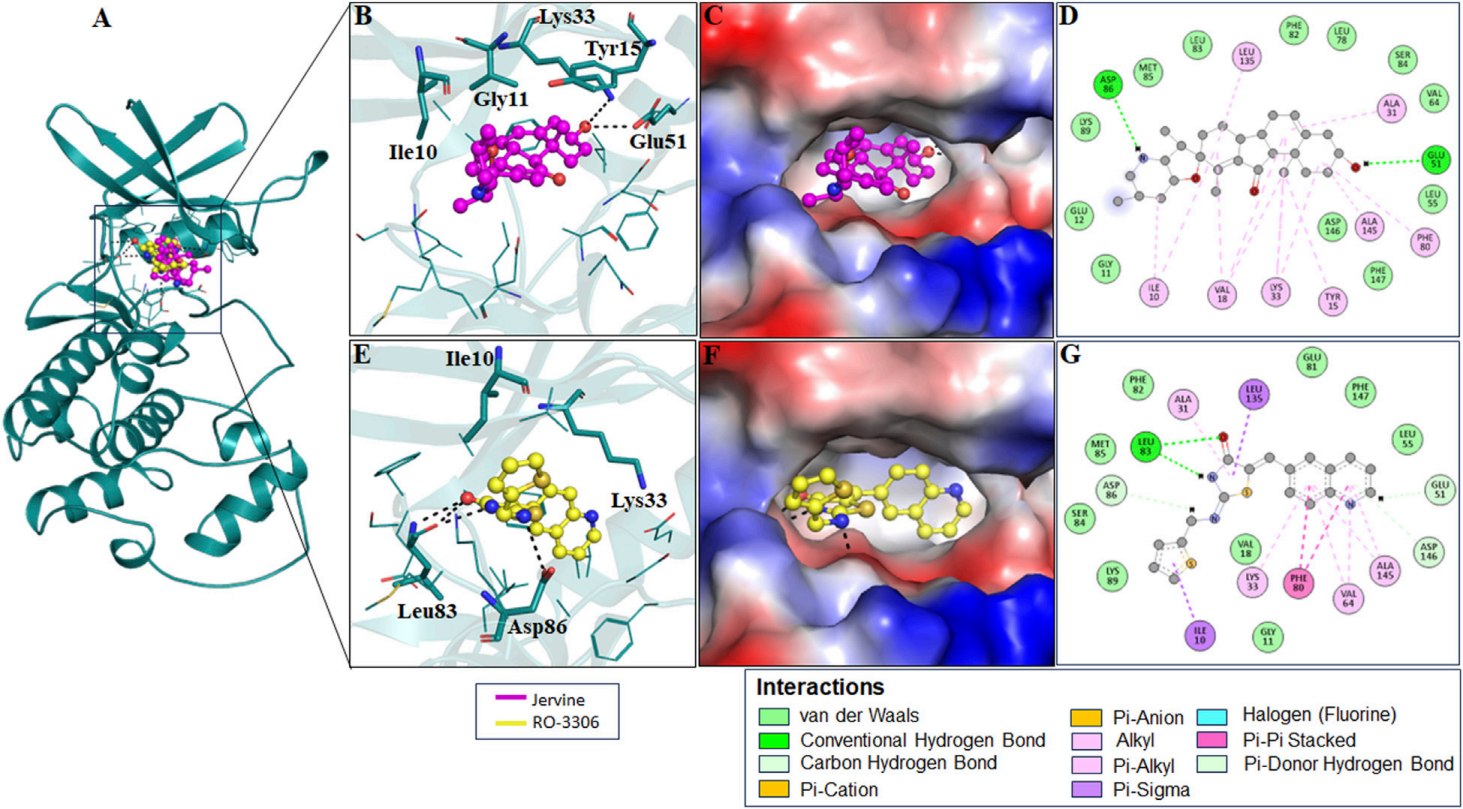
Structural representation of CDK1 with ligands. Interaction analysis of CDK1 with Jervine and RO-3306. **(A)** 3D interaction of CDK1 with Jervine and RO-3306. **(B)** Enlarged view depicting the key amino acid residues involved in the interaction between CDK1 and Jervine. **(C)** Surface electrostatic potential view of CDK1 with Jervine. **(D)** 2D interaction diagram showing hydrogen bonds and hydrophobic interactions between CDK1 and Jervine. **(E)** Enlarged view depicting the key amino acid residues involved in the interaction between CDK1 and RO-3306. **(F)** Surface electrostatic potential map illustrating the binding interface of CDK1 with RO-3306. **(G)** 2D interaction diagram showing hydrogen bonds and hydrophobic interactions between CDK1 and RO-3306.

The interaction analysis of Jervine, with AURKB and CDK1 was conducted along with respective inhibitors VX-680 and RO-3306 to elucidate the probable binding sites of compounds. The in-depth analysis revealed that Jervine interacted with Asp200, the catalytic site residue of AURKB, by forming a direct hydrogen bond. The interaction analysis showed that VX-680 formed only non-covalent interactions with the active site and residues surrounding the ATP-binding pocket of AURKB (Figures 1A–E). A comparable interaction profile was observed between Jervine and CDK1, in contrast to RO-3306. Notably, Jervine established multiple hydrogen bonds with residues of CDK1, including Glu51 and Asp86 (Figures 2A–E). In contrast, the approved CDK1 inhibitor did not exhibit hydrogen bond formation in the analysis. Additionally, Jervine engaged in several non-covalent interactions with substrate-binding residues of CDK1. Detailed interaction data is presented in Table 3.

**TABLE 3:** Interacting residues of AURKB and CDK1 with Jervine and respective inhibitors.

Types of interaction	Protein-drug complex			
	AURKB-Jervine	AURKB-VX-680	CDK1-Jervine	CDK1-RO-3306
Conventional ydrogen bond	Asp200	Cys258	Asp86, Glu51	Leu83
Van der waals bond	Trp257, Pro203, Ile201, His198, Val254, Ile285, Ser275, Glu274, Thr281	Asp200, His250, Lys202, Pro272, Phe273, Thr281, Ser275, Glu274, Val254, Arg315, Leu228.	Met85, Ser84, leu83, Phe82, leu78, Val64, Leu55, Asp146, Phe147, Gly11, Glu12, Lys89.	Phe82, Glu81, Phe147, Leu55, Gly11, Val18, Lys89, Ser84, Met85.
Alkyl/Pi-Alkyl	Phe273, Pro272, Val261, Cys258, Lys202, Pro271	Val261, Pro271, Ile285	Leu135, Ala31, Ala145, Phe80, Lys33, Val18, Tyr15, Ile10.	Ala31, Ala145, Val64, Lys33
Carbon-hydrogen bond	His250	—	—	Asp86, Glu51, Asp146,
Pi sigma	—	—	—	Ile10, Leu135
Pi-Pi stacked			—	Phe80

### 3.6 MD simulation studies

MD simulations are essential in understanding the structural details and alterations in the dynamics and behaviour of different biomolecular systems, including protein-ligand complexes, at the atomic level. This comprehensive tool also provides deeper insights into the thermodynamics, kinetics and stability of biological molecules and associated ligands (Khan et al., 2019). MD simulations offer a detailed outlook into structural dynamics, addressing complexities often challenging to capture through experimental approaches (Lobanov et al., 2008; Salsbury Jr, 2010). To understand the conformational dynamics, four systems of docked complexes, including AURKB-Jervine, AURKB-VX-680, CDK1-Jervine, and CDK1-RO-3306, were subjected to an MD simulation of 300 ns to elucidate the stability during the designated time frame. A 300 ns simulation was performed, as this duration is generally sufficient to ensure convergence of structural and energetic parameters in protein-ligand complexes, as reported in previous studies on similar systems. This timescale enabled us to capture stable binding interactions and monitor conformational changes of the AURKB and CDK1 complexes. Furthermore, our choice is consistent with literature reports, where comparable or shorter simulation windows (50 ns–200 ns) have been successfully employed to investigate related kinase-ligand systems (Habib et al., 2024b; Jairajpuri et al., 2025).

### 3.7 Analysis of structural dynamics

The simulation extracted structural parameters, including root mean square deviation (RMSD), root mean square frequency, radius of gyration (*R*g), and solvent-accessible surface area (SASA) were calculated. These trajectories provide information on the protein’s changing conformational state upon the ligand’s invasion (Table 4).

**TABLE 4:** Calculated MD simulation parameters obtained after a 300 ns simulation.

System	RMSD (nm)	RMSF (nm)	*R*g (nm)	SASA (nm^2^)	#Intra H-bond
AURKB	0.416012	0.24208	2.00617	154.479	165
AURKB-Jervine	0.388152	0.14856	1.97728	150.224	173
AURKB-VX-680	0.456133	0.209199	1.98789	156.336	165
CDK1	0.21723	0.112584	2.02746	152.334	190
CDK1-Jervine	0.288654	0.135309	2.06300	158.753	185
CDK1-RO-3306	0.258938	0.115183	2.05533	155.081	184

**FIGURE 3:**
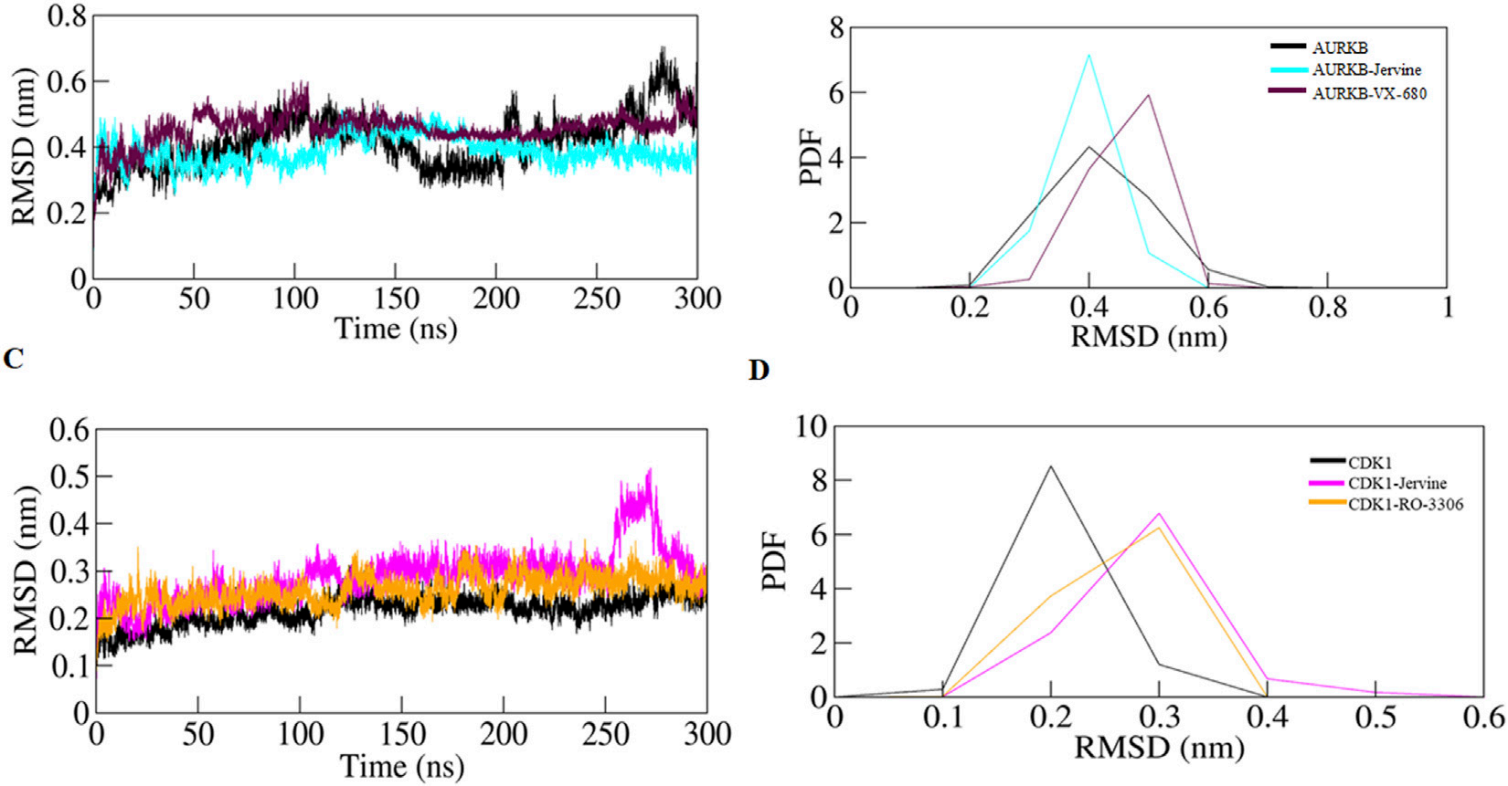
RMSD plot of AURKB and CDK1 with ligands as a function of time **(A)** free AURKB, AURKB-Jervine and AURKB-VX-680, **(B)** PDF of RMSD plot, **(C)** free CDK1, CDK1-Jervine and CDK1-RO-3306, **(D)** PDF of RMSD plot.

RMSD, a crucial trajectory of MD simulations, provides valuable insights into the extent of protein flexibility, folding, and overall dynamics upon ligand binding. This parameter quantifies the average atomic displacement of protein backbone atoms over time relative to a reference structure, typically the initial conformation. RMSD values obtained for native AURKB, AURKB-Jervine, and AURKB-VX680 were found to be 0.416 nm, 0.388 nm, and 0.456 nm, respectively. RMSD of AURKB-Jervine was found to be low compared to free AURKB and AURKB-VX-680 complex. Interestingly, the RMSD of the AURKB-Jervine complex was slightly less than that of native AURKB and the AURKB-VX-680 complex, indicating the formation of a more stable complex. However, the observed changes were minimal and stabilised by the end of the simulation, likely due to the initial positioning of Jervine within the AURKB binding pocket. These findings suggest that the AURKB-Jervine complex exhibits higher structural stability over the simulation time than the native protein and AURKB complexed with VX-680 (Figures 3A,B).

Similarly, for native CDK1, CDK1-Jervine and CDK1-RO-3306, the RMSD values were 0.217 nm, 0.288 nm and 0.258 nm, respectively. Here, it was observed that the CDK1-Jervine complex has an RMSD value similar to that of native CDK1 and the CDK1-RO-3306 complex. These findings indicate that binding of Jervine to either AURKB or CDK1 does not trigger significant structural changes and deviations that could destabilise the protein. It can be concluded that Jervine gets well accommodated in the binding pocket of both enzymes without compromising overall structural integrity (Figures 3C,D).

**FIGURE 4:**
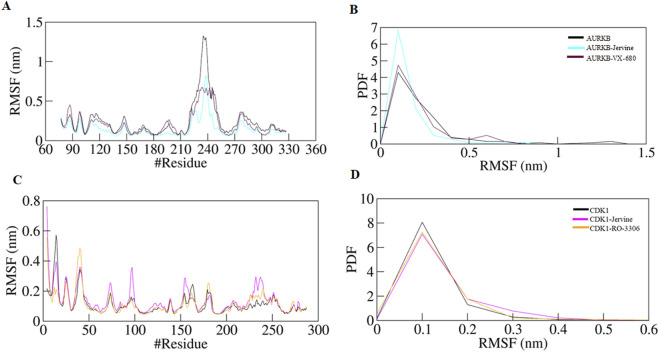
RMSF plot of AURKB and CDK1 with ligands as a function of time **(A)** free AURKB, AURKB-Jervine and AURKB-VX-680, **(B)** PDF of RMSF plot, **(C)** free CDK1, CDK1-Jervine and CDK1-RO-3306, **(D)** PDF of RMSF plot.

The following structural parameter investigated was RMSF, a key metric used to assess the flexibility of individual amino acid residues in a protein structure. It provides information on localised structural dynamics upon ligand binding, unlike RMSD, which indicates overall structural deviation. RMSF projects residue-specific fluctuations over the entire simulation. RMSF values obtained for native AURKB, AURKB-Jervine and AURKB-VX-680 were 0.242 nm, 0.148 nm and 0.209 nm. It was noted that Jervine binding resulted in a slight deviation and decrease in RMSF compared to native AURKB (Figures 4A,B). This indicates Jervine-induced structural stabilisation of AURKB, most likely in the regions with reduced flexibility (Figure 2C). The RMSF of native CDK1, CDK1-Jervine and CDK1-RO-3306 were 0.112 nm, 0.135 nm and 0.115 nm, respectively, and were slightly higher than that of native CDK1 and the CDK1-RO-3306 complex (Figures 4C,D). However, these changes were found to be non-significant as the equilibrium was achieved by the end of the simulation.

**FIGURE 5:**
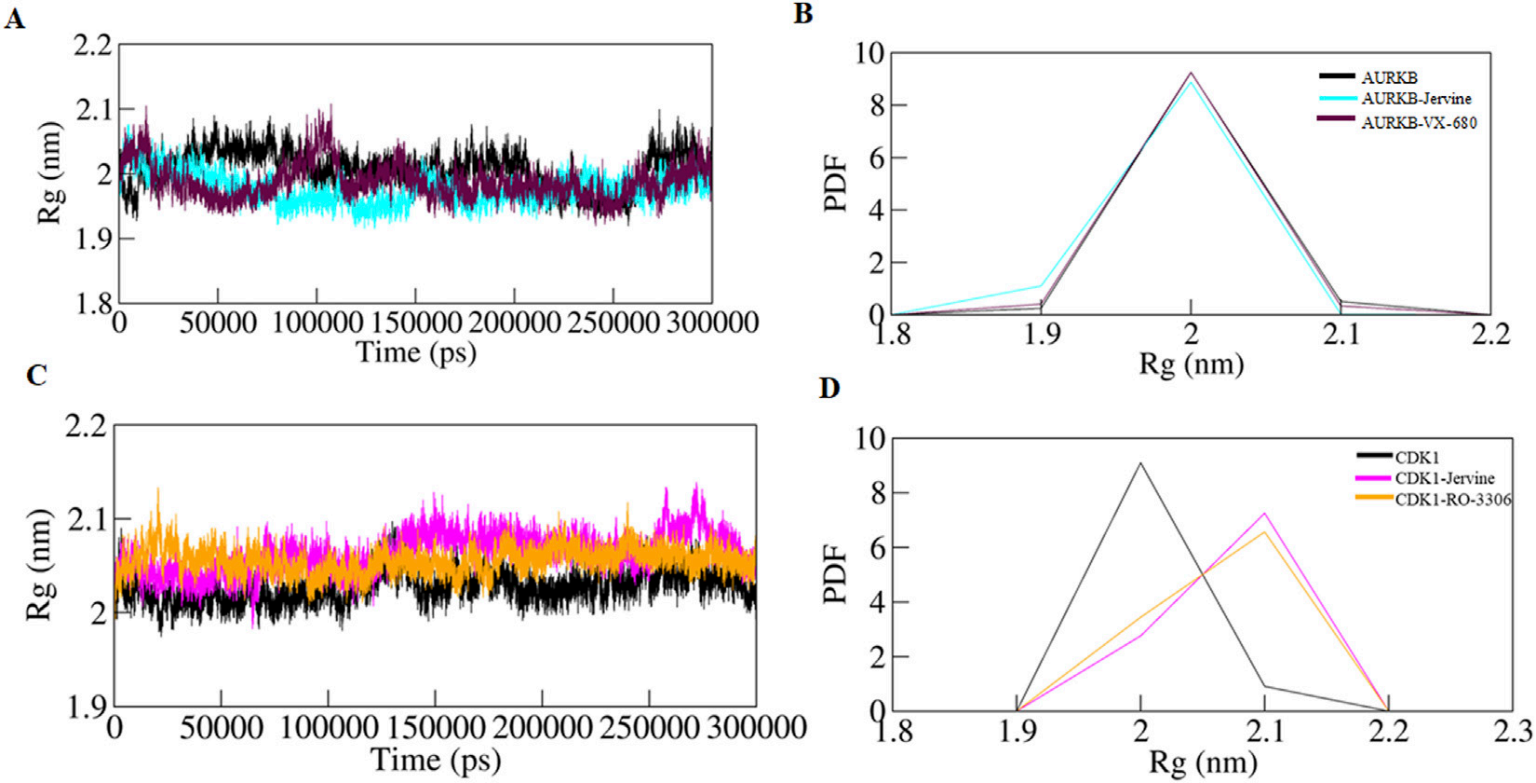
Time evolution of radius of gyration **(A)** free-AURKB, AURKB-Jervine and AURKB-VX-680, **(B)** PDF of *R*g plot, **(C)** free CDK1, CDK1-Jervine and CDK1-RO-3306, **(D)** PDF of *R*g plot.

*R*g was calculated to further understand the structural compactness of AURKB and CDK1, which provided details about the folding pattern and structural integrity in the presence of ligands. The *R*g of native AURKB, AURKB-Jervine, and AURKB-VX-680 complexes were 2.006 nm, 1.977 nm, and 1.987 nm, respectively (Figures 5A,B). The results indicate that Jervine binding induces a more compact and stable AURKB conformation than the native state, reflecting enhanced structural integrity and a stabilising effect of Jervine on the AURKB structure. Similar *R*g findings were obtained for native CDK1, CDK1-Jervine, and CDK1-RO-3306 complexes, which were 2.027 nm, 2.063 nm, and 2.055 nm, respectively. Here, the structural compactness of CDK1 was almost similar to that of the native state and CDK1-RO-3306 (Figures 5C,D).

**FIGURE 6:**
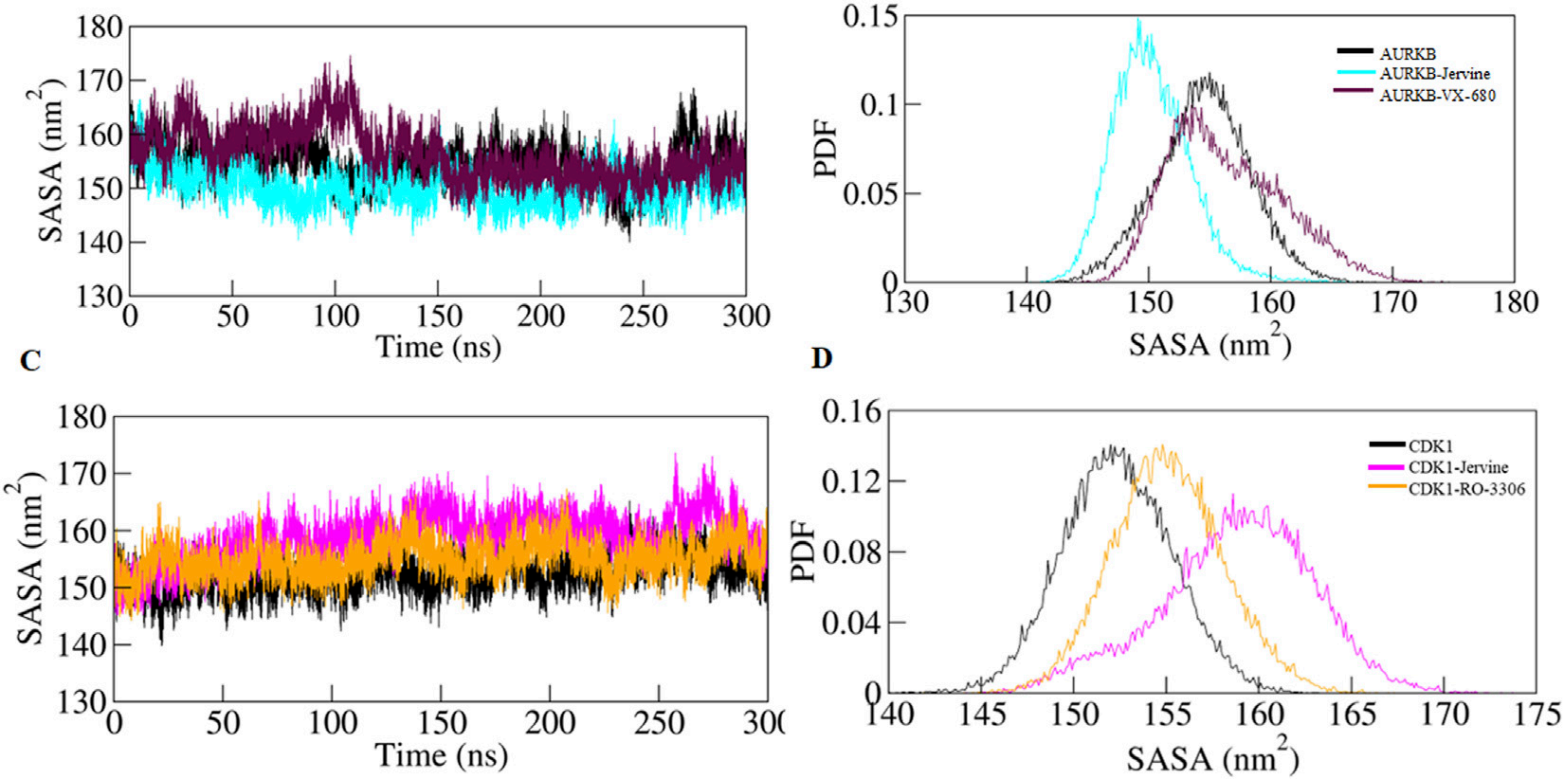
SASA plot as a function of time **(A)** free-AURKB, AURKB-Jervine and AURKB-VX-680, **(B)** PDF of SASA plot, **(C)** free CDK1, CDK1-Jervine and CDK1-RO-3306, **(D)** PDF of SASA plot.

To further validate the *R*g findings, SASA was also determined for all the systems. SASA is a key parameter of MD simulation studies that gives information on changes in the solvent-accessible surface area of a protein upon binding ligands. Changes in SASA values depict structure alterations due to exposure of hydrophobic and hydrophilic residues to the solvent environment. The SASA of the native AURKB, AURKB-Jervine, and AURKB-VX-680 complex was found to be 154.47 nm^2^, 150.22 nm^2^, and 156.33 nm^2^, respectively (Figures 6A,B).

This shows that the binding of Jervine reduced SASA compared to native AURKB and had better compactness than VX-680. On the other hand, the SASA of native CDK1, CDK1-Jervine and CDK1-RO-3306 was found to be 152.33 nm^2^, 158.75 nm^2^, and 155.08 nm^2^. However, in CDK1, binding of Jervine slightly enhanced the SASA values, which were non-significant. These findings also align with other trajectories, including RMSD, RMSF and *R*g. Jervine mostly enhanced the overall stability parameters of AURKB, while maintaining structural integrity as in CDK1 similar to native state (Figures 6C,D). The replicas of the simulation have been provided in Supplementary Figures S1–S3 and Supplementary Table S6.

### 3.8 Hydrogen bond analysis

**FIGURE 7:**
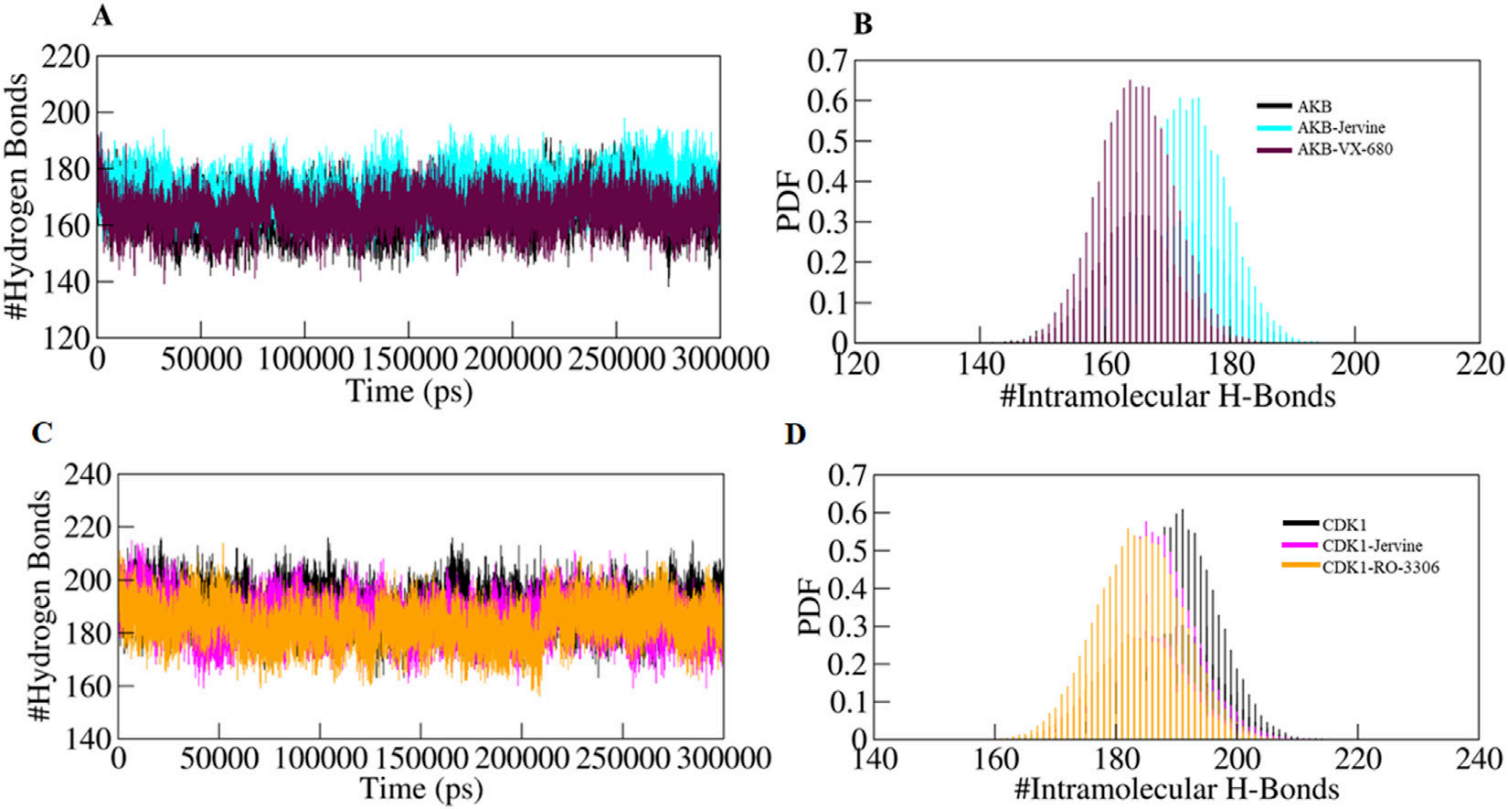
Intra-molecular hydrogen bonds **(A)** free-AURKB, AURKB-Jervine and AURKB-VX-680, **(B)** PDF plot, **(C)** free CDK1, CDK1-Jervine and CDK1-RO-3306, **(D)** PDF plot.

Intramolecular hydrogen bonds and hydrophobic interactions are crucial in stabilising the structure of the protein, thereby facilitating its 3D conformation. Analysis of these bonds gives essential insights into structural compactness and dynamics. Intramolecular hydrogen bonds exist in the protein itself, stabilising the structural elements. The formation of intramolecular hydrogen bonds in AURKB and CDK1 was observed over time using MD trajectories. The formation of intramolecular hydrogen bonds in the unbound state of AURKB and CDK1, and after binding of Jervine and with their respective inhibitors, was noted. In the case of AURKB, the number of intramolecular bonds increases upon binding of Jervine with AURKB (173) compared to the native protein (165) and complexed with VX-680 (165) (Figures 7A,B). However, in CDK1, the number of intramolecular bonds was found with Jervine (185) to be less than that of the native CDK1 (190) complexed with RO-3306 (184) (Figures 7C,D). These findings indicate that both proteins retained structural compactness after binding their respective ligands.

**FIGURE 8:**
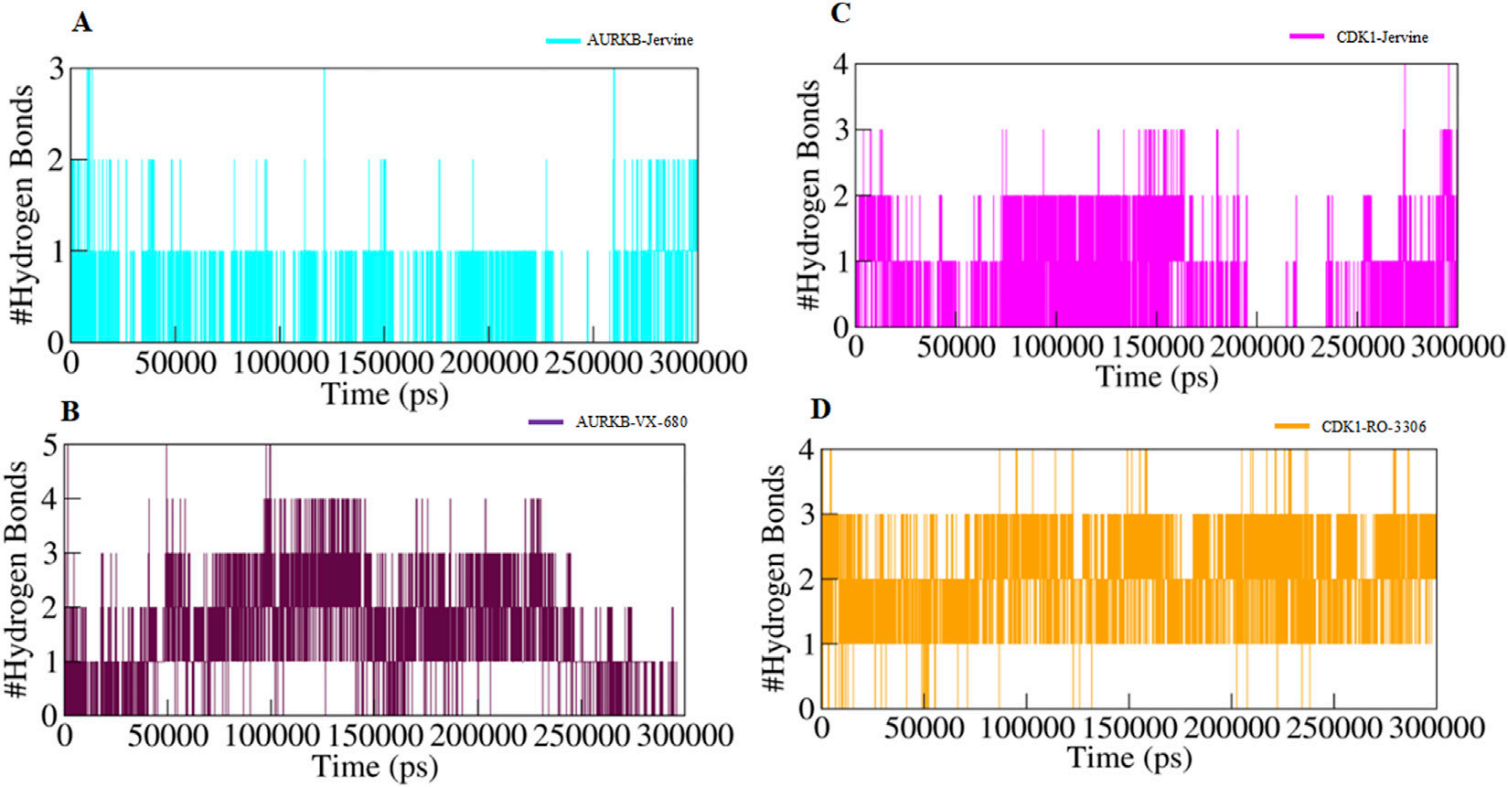
Intermolecular hydrogen bonds **(A)** AURKB-Jervine, **(B)** AURKB-VX-680, **(C)** CDK1-Jervine, **(D)** CDK1-RO-3306.

In addition, intermolecular hydrogen bonding was also analysed to identify the bonds formed between protein and ligand. These bonds play a crucial role in stabilising the protein-ligand complex during simulations. The number of hydrogen bonds formed between AURKB and CDK1 with their ligands is provided in Figure 8.

### 3.9 Secondary structure analysis

Parameters, including *α*-helix, *β*-sheet, and turns, were plotted throughout the simulation to examine how the secondary structure changes upon ligand binding. Secondary structure analysis provides information about protein’s structural integrity and conformational transitions upon ligand binding. Investigating the changes in *α*-helices, *β*-sheets, and other structural elements with time can assess the changes in the native structure of the protein and partial or global unfolding. The binding of Jervine to AURKB slightly reduced the coil content and increased the *β*-sheets and *α*-helix compared to the native protein but was almost similar to VX-680. The other structural elements were unchanged in all three systems (Supplementary Figure S4). However, in CDK1, Jervine binding did not cause significant alterations in any structural element and was similar to native CDK1 and CDK1-RO-3306 complex (Supplementary Figure S4; Supplementary Table S7). These findings project the formation of a stable complex in all systems without any conformational changes in AURKB and CDK1.

### 3.10 Principal component analysis

Principal Component Analysis (PCA) is a statistical tool for MD simulations that captures and analyses dominant atomic motions and conformational alterations in proteins upon ligand binding (Mohammad et al., 2020a; Mohammad et al., 2020b). Proteins do the collective motion of their atoms to perform specific functions. The dynamics of a protein can be illustrated through its phase space behaviour. Eigenvalues were extracted through the covariance matrix and the principal components (PCs) while using the *gmx anaeig* and *gmx covar* tools to investigate the principal motion directions in the essential subspace (Anjum et al., 2022). The eigenvalues corresponding to each eigenvector (EV) were extracted to indicate the direction of motion in the essential phase space. MD simulations generate large amounts of data, representing the atomic coordinates of a biomolecular system over time. PCA was performed using the essential dynamics approach to examine the conformational sampling of both the targets in their native form as well as in complex form AURKB, AURKB-Jervine, AURKB-VX-680, CDK1, CDK1-Jervine and CDK1-RO-3306. This analysis facilitated the exploration of collective movements and conformational changes of the target protein based on the simulated trajectories.

**FIGURE 9:**
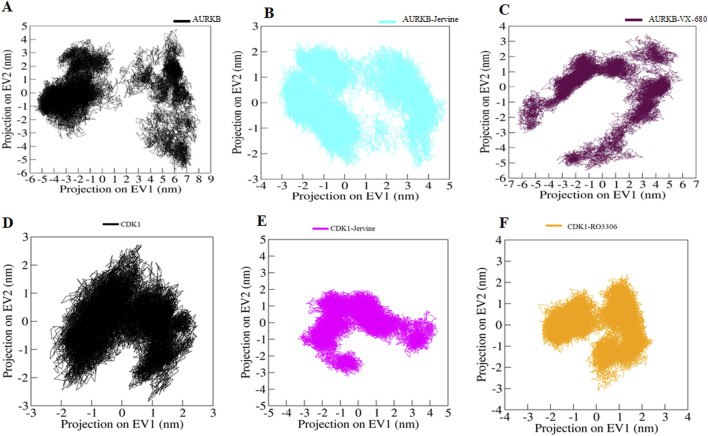
Principal component analysis. 2D projections of trajectories on eigenvectors showing different projections of **(A)** free-AURKB, **(B)** AURKB-Jervine, **(C)** AURKB-VX-680, **(D)** free-CDK1, **(E)** CDK1-Jervine, **(F)** CDK1-RO-3306.

The conformational sampling was projected onto the alpha carbon atoms (C^α^) in the six systems. The results indicated that AURKB and its complexes, both of which shared the same subspace, had a smaller subspace of flexibility in the case of AURKB-Jervine, decreasing the subspace flexibility of AURKB-VX-680 (Figures 9A–C). On the other hand, CDK1-Jervine and CDK1-RO-3306 complexes shared the same subspace as the unbound CDK1. However, a slightly smaller subspace of flexibility was seen in the case of the CDK1-RO-3306 complex, which decreased the flexibility of CDK1-Jervine (Figures 9D–F). Overall, both targets form stable complexes with Jervine and show similar results with their known inhibitors, making Jervine a potential therapeutic agent for cancer (Sulaimani et al., 2025)

### 3.11 Free energy landscapes

**FIGURE 10:**
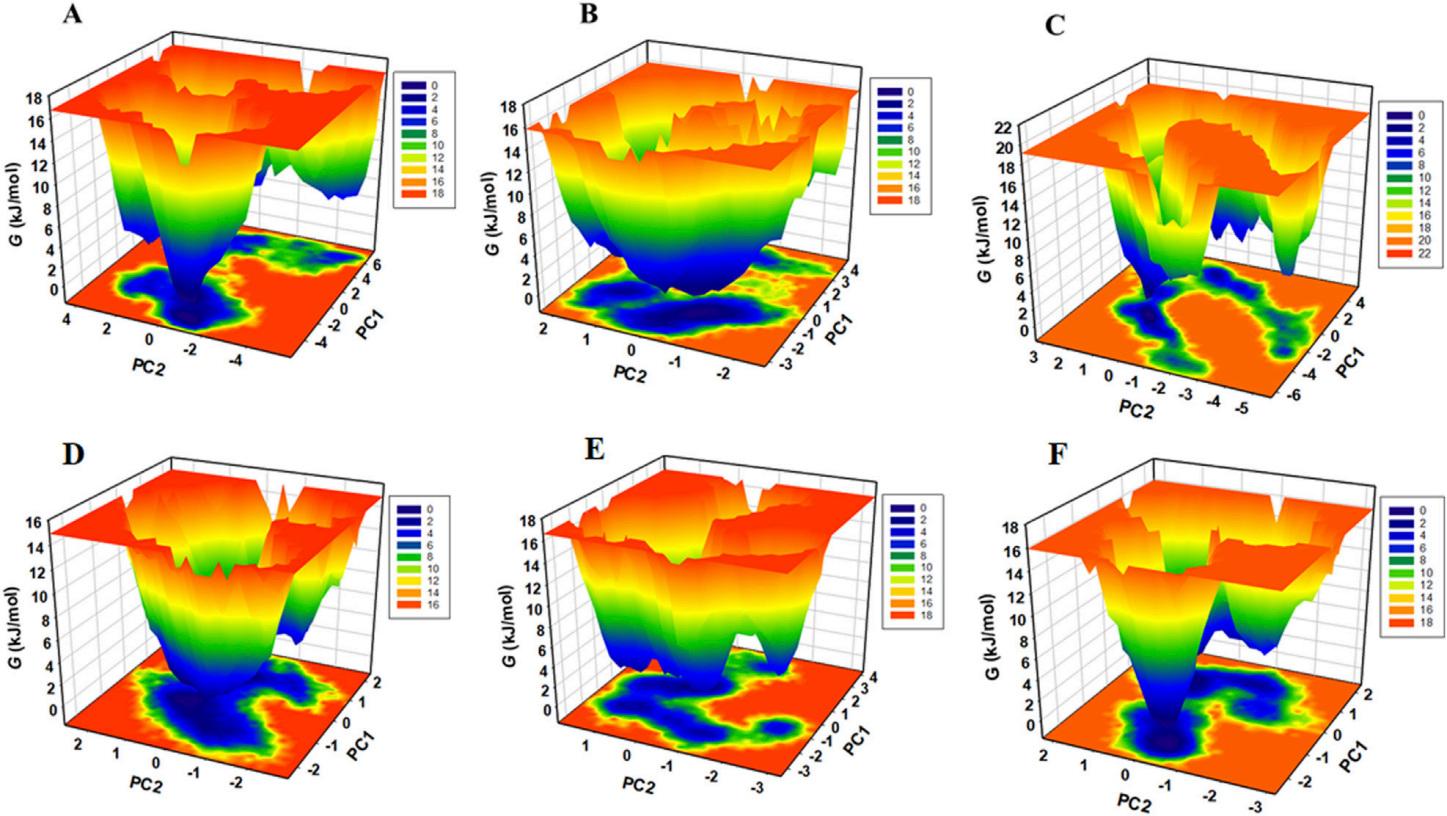
Gibbs free energy landscape generated by projecting the principal components, PC1 and PC2, during MD simulation **(A)** free-AURKB, **(B)** AURKB-Jervine, **(C)** AURKB-VX-680, **(D)** free-CDK1, **(E)** CDK1-Jervine, **(F)** CDK1-RO-3306.

Free energy landscapes (FELs) often depict the energetically favored conformations and transitions between different states of a biomolecular system. FELs were utilised to assess the stability of protein molecules and their complexes with ligands in a solvent environment. FELs offer valuable insights into protein folding, denaturation, energy minima, and conformational landscapes (Habib et al., 2024a). Using the first two PCs, we constructed FELS to analyse the energy minima and conformational dynamics of unbound CDK1 and AURKB and their respective ligand-bound complexes. The resulting FEL plots for CDK1 and AURKB in both unbound and bound states are presented in Figures 10A–C.

The FEL of unbound AURKB exhibited multiple basins with identifiable global minima. In contrast, the AURKB-Jervine complex displays a single large basin with a prominent global minimum, suggesting a more stable conformational state than the AURKB-VX-680 complex, which shows relatively less stability (Rathi et al., 2025). The FEL of unbound CDK1 showed a dominant, well-defined global minimum, indicating a stable native conformation (Figures 10D–F). Upon binding with Jervine and RO-3306, CDK1 adopts multiple conformational states, as suggested by the presence of several energy basins, although a global minimum remains. This shift in the energy landscape implies that ligand binding introduces moderate perturbations in the conformational phase space of CDK1. A similar pattern is observed for AURKB and its complexes.

In all FELs, deeper blue regions correspond to lower-energy, near-native conformations. Despite the conformational changes upon ligand binding, the overall structural integrity of both AURKB and CDK1 was maintained throughout the simulations. This indicates that binding of Jervine and the respective known inhibitors does not lead to protein unfolding.

### 3.12 MMPBSA analysis

MMPBSA analysis was carried out to estimate the binding free energy of the AURKB and CDK1 protein-ligand complexes using gmx_MMPBSA module in GROMACS. Binding energy is a thermodynamic parameter that shows the change in energy associated with the formation of a bond, and it can be used to describe the strength of the interactions between a ligand and a protein. The binding free energy components, including van der Waals interactions and their corresponding average standard deviations, as obtained from the MM/PBSA analysis, are summarised in Table 5. The results demonstrate that all AURKB-ligand complexes exhibit favourable binding free energies, indicating the formation of stable complexes. Among them, the AURKB-Jervine complex showed the highest binding affinity (−15.51 kJ/mol), closely comparable to the control AURKB-VX-680 complex (−13.99 kJ/mol), suggesting enhanced stability. Similarly, the CDK1-Jervine complex exhibited a strong binding affinity (−25.34 kJ/mol), nearly equivalent to RO-3306 (−25.25 kJ/mol), further indicating the formation of a stable complex.

**TABLE 5:** MMPBSA calculations of binding free energy for protein-ligand complexes.

Complex	ΔVDWAALS	ΔE_EL_	ΔEPB	ΔE_NPOLAR_	ΔGGAS	ΔGSOLV	Standard deviation	${\Delta G}_{\text{Total}\;\left(\text{kJ}/\text{mol}\right)}$
AURKB-Jervine	−21.24	−15.14	23.64	−2.77	−36.38	20.87	1.75	−15.51
AURKB-VX-680	−21.23	−6.87	16.76	−2.65	−28.10	14.11	3.53	−13.99
CDK1-Jervine	−38.05	−20.03	36.99	−4.24	−58.09	32.75	7.53	−25.34
CDK1-RO-3306	−44.08	−26.59	49.52	−4.10	−70.67	45.42	4.12	−25.25

## 4 Discussion

Natural products have gained significant attention for decades due to their excellent pharmacological and therapeutic properties (Domingo-Fernández et al., 2024). Therefore, exploration of phytochemicals against molecular targets holds great promise (Rudzińska et al., 2023). This study screened IMPPAT, a natural product database, to determine potent dual-targeting inhibitors against AURKB and CDK1. The study implied a multi-tier approach, including high-throughput screening, ADMET analysis, PASS evaluation, MD simulations and MMPBSA to identify common inhibitor. Drug-likeliness and pharmacokinetic properties were also compared with known inhibitors of AURKB and CDK1, VX-680 and RO3306, respectively. A detailed docking analysis revealed that Jervine is a common compound among the top 50 hits of AURKB and CDK1, showing a high binding affinity and appreciable ligand efficiency for both enzymes. Jervine exhibited a higher binding affinity than control inhibitors (VX-680 and RO-3306) of AURKB and CDK1. This indicates a more stable complex formation between proteins and Jervine than control drugs.

Jervine was evaluated for physicochemical and ADMET properties along with control drugs. ADMET analysis revealed that Jervine exhibited better GI absorption than control drugs and non-mutagenic features. Jervine exhibited high permeability across the blood-brain barrier, suggesting its potential utility in treating central nervous system (CNS) related disorders. However, its use in non-CNS models may pose a risk of off-target effects or adverse outcomes due to unintended CNS exposure. Among ADMET parameters, Jervine was found to be a substrate of OCT2, which indicates its smooth renal clearance. Interestingly, none of the control drugs were OCT2 substrates, which signifies a possibility of systemic accumulation of these drugs, potentially causing toxicity. The natural compound Jervine surprisingly demonstrated superior ADMET features compared to selective reference drugs against AURKB and CDK1, highlighting its potential as a safer and more effective alternative with minimal adverse effects.

PASS analysis, an *in silico* tool that predicts the biological and therapeutic role of any drug based on its chemical structure, was conducted. It was found that, like VX-680 and RO3006, Jervine also depicted anti-neoplastic properties. This indicates that Jervine might share structural similarities with compounds or drugs that are well-reported to inhibit tumor growth. Moreover, this also implies that Jervine harbours strong anti-cancer potential through modulation of key cellular processes critical for cancer development and progression (Chen et al., 2020; Lei and Huo, 2020).

After thoroughly evaluating biological properties, ADMET, and pharmacokinetic parameters, an in-depth computational analysis of the binding mechanism of Jervine with AURKB and CDK1 was conducted. 2D-interaction analysis depicted the formation of hydrogen bonds between Jervine and the ATP-binding pocket of AURKB. Also, Jervine formed hydrogen bonds with the catalytic site residue Asp200 of AURKB, which signifies its higher anti-kinase potential. Similar findings were also observed in the CDK1-Jervine complex, where Jervine interacted with important residues of CDK1 through several covalent and non-covalent interactions. Compared to VX-680, Jervine formed covalent bonds with key residues of AURKB catalytic core, whereas VX-680 exhibited only non-covalent interactions. Compounds showing non-covalent interactions with targets often exhibit a reversible binding mode, dynamically allowing frequent association and dissociation (Bandyopadhyay and Gao, 2016). However, compounds that bind covalently show prolonged target interactions, thereby increasing potency, but at the same time might impose serious off-target effects (Bandyopadhyay and Gao, 2016). Hence, computational tools can be used to predict these interaction mechanisms, aiding in the drug development process.

MD simulations were performed for each drug-protein complex further to investigate stability, conformational dynamics, and interaction patterns (Hollingsworth and Dror, 2018). This technique gives detailed insights into the binding mode, key residue flexibility, and conformational adaptations in the protein upon ligand binding, providing predictions on drug efficacy and specificity. In the case of AURKB, the simulation parameters, including RMSD, RMSF, *R*g, and SASA, cumulatively indicated that AURKB-Jervine was more stable than the AURKB-VX-680 complex. Compared to the control, decreased RMSD and RMSF values in the AURKB-Jervine complex reflected lesser structural deviations and consistent interactions with the protein. Additionally, lower *R*g indicated the formation of a compact complex of AURKB and Jervine, whereas reduced SASA specified a decrease in solvent exposure.

However, the changes were non-significant but provided a better understanding of enhanced AURKB-Jervine stability compared to the VX-680 complex. However, in the case of CDK1, the simulation parameters RMSD and RMSF were negligibly higher in the CDK1-Jervine complex compared to RO-3360 and the native protein. Similarly, the *R*g and SASA values were also higher. These differences were, however, considered non-significant. This further indicates that the stability parameters of the CDK1-Jervine complex were almost similar to those of native CDK1 and the CDK1-RO-3306 complex, which means that Jervine did not trigger any structural perturbations or instability when bound to CDK1, and stability parameters were similar to those of a known CDK1 inhibitor.

Changes in secondary structure can disrupt the stability and functionality of proteins (Koop et al., 2020). Therefore, this study calculated the secondary structure content of all drug-ligand complexes using MD simulations. When AURKB interacted with Jervine, a slight decrease in amino acid residues forming coils was found compared to the native protein. This might occur due to the initial fitting of the ligand within the catalytic core of the enzyme. However, in the case of CDK1, the secondary structure content remained almost the same. These findings indicate that Jervine binding did not trigger any structural perturbations in either of the enzymes.

The essential dynamics analysis, conducted through PCA and FEL mapping, offered valuable insights into the conformational stability and dynamic behavior of AURKB and CDK1 and their respective complex with the selected common compound, Jervine. The analysis revealed that Jervine maintains the structural integrity of both target proteins while inducing favorable conformational states, indicating strong and stable interactions. Lastly, MMPBSA analysis was conducted to determine the binding free energy between all drug-protein complexes after molecular docking and MD simulations. This analysis quantitatively measures binding affinity based on molecular mechanics and solvation energies. AURKB-Jervine complex showed a more negative binding affinity than the AURKB-VX-680 complex. It can be concluded that Jervine showed stronger and more stable binding with AURKB than the reference drug. In addition, Jervine had more favourable interactions with the active pocket of AURKB, better potency, and therapeutic efficacy than VX-680. However, the binding affinities from the MMPBSA analysis of the CDK1-Jervine complex were almost similar to those of the CDK1-RO-3306 complex. This implies that Jervine has a comparable stability, efficacy, and therapeutic potential as RO-3306 against CDK1.

The findings of this study collectively underscore the potential of Jervine as a dual inhibitor, capable of effectively targeting both AURKB and CDK1. While our findings highlight a possible mechanistic link, further extensive and in-depth *in vitro* and *in vivo* studies are necessary to explore the downstream molecular pathways and define the mode of action of Jervine. In addition, *in vivo* models are needed to investigate the pharmacodynamics and pharmacokinetic properties of Jervine comprehensively. This approach opens avenues for further optimisation and structural modification of the Jervine backbone, or screening of related steroidal alkaloids may yield safer analogues with retained inhibitory activity. The promising results of this study provide a robust foundation for further experimental validation and preclinical development, supporting the continued exploration of Jervine as a viable therapeutic candidate for cancer treatment.

## 5 Conclusion

This study used an integrated computational approach to systematically identify Jervine, a compound from the IMPPAT database, as a promising drug candidate with dual kinase targeting properties against AURKB and CDK1. High-throughput virtual screening, ADMET analysis, drug-likeliness, and PASS evaluation depicted Jervine as a strong drug candidate against reference compounds VX-680 and RO-3306. The findings of molecular docking revealed higher binding affinities of Jervine towards AURKB and CDK1, indicating strong interactions and structural stability. ADMET analysis revealed appreciable and advantageous pharmacokinetic parameters in Jervine compared to control drugs. Based on the structure-activity relationship, Jervine exhibited anti-neoplastic potential similar to VX-680 and RO-3306.

Finally, MD simulation studies validated the stability of Jervine and protein complexes, particularly with AUKRB. Also, binding of Jervine did not trigger any structural perturbations in either target protein. Free energy calculations demonstrated favourable binding energetics of Jervine-AURKB complex compared to VX-680, while demonstrating comparable stability with CDK1 relative to RO-3306. Hence, the overall findings of this study indicate that Jervine is a potent, highly stable, and pharmacologically active drug molecule that could offer significant promise in cancer therapeutics *via* dual targeting of cell cycle kinases.

## 6 Limitations

Overall, the study provides a detailed analysis of drug identification but has limitations, such as exclusive reliance on computational approaches for identifying dual inhibitors against AURKB and CDK1. The study must be further validated on experimental models with comprehensive clinical validations. Future work should include in-depth *in vitro* and *in vivo* studies to confirm Jervine’s dual inhibitory potential towards AURKB and CDK1 and assess its translational feasibility as an anticancer therapeutic. Also, extensive pharmacological profiling, structure optimisation, or identification of design and development of safer analogues is crucial before considering its therapeutic potential.

## Data Availability

All data supporting the findings of this study are available within the paper. The phytochemicals and protein structures analyzed are publicly available from IMPPAT (https://cb.imsc.res.in/imppat/) and the RCSB Protein Data Bank (https://www.rcsb.org/structure/4AF3 and https://www.rcsb.org/structure/6GU2).

## Abbreviations

ADMET, Absorption, distribution, metabolism, excretion, and toxicity; ATP, Adenosine tri-phosphate; AURKB, Aurora kinase B; CHARMM, Chemistry at Harvard Macromolecular Mechanics; CDK1, Cyclin dependent kinase 1; CPC, Chromosomal passenger complex; CNS, Central nervous system; IMPPAT, Indian medicinal plants phytochemistry and therapeutics; MMPBSA, Molecular mechanics/Poisson-Boltzmann surface area; PASS, Prediction of activity spectra for substances; MD, Molecular dynamics; Rg, Radius of gyration; RMSD, Root mean square deviation; RMSF, Root mean square frequency; SASA, Solvent accessible surface area.
