# Supplementary material for: Structure-based identification of Jervine as a potent dual-targeting inhibitor of cell cycle kinases
Source: Front Pharmacol. 2025 Nov 21;16:1662556. doi: 10.3389/fphar.2025.1662556 (PMC12678241; doi:10.3389/fphar.2025.1662556)
Supplement: Supplementary file 1 [file Supplementaryfile1.docx]

**Supplementary Data**

**Table S1**: Selected hits and a control molecule with their docking scores with AURKB

| **S. No.** | **Compound Name** | **Affinity (kcal/mol)** | **pKi** | **Ligand Efficiency (kcal/mol/non-H atom)** |
| --- | --- | --- | --- | --- |
|  | IMPHY006230 | −10.6 | 7.77 | 0.4417 |
|  | IMPHY000086 | −10.3 | 7.55 | 0.4478 |
|  | IMPHY012046 | −10.1 | 7.41 | 0.3885 |
|  | IMPHY008970 | −9.9 | 7.26 | 0.4125 |
|  | IMPHY010181 | −9.9 | 7.26 | 0.3094 |
|  | IMPHY001309 | −9.7 | 7.11 | 0.2694 |
|  | IMPHY014146 | −9.7 | 7.11 | 0.3031 |
|  | IMPHY007425 | −9.7 | 7.11 | 0.485 |
|  | IMPHY011452 | −9.6 | 7.04 | 0.32 |
|  | IMPHY002427 | −9.5 | 6.97 | 0.3654 |
|  | IMPHY016907 | −9.5 | 6.97 | 0.475 |
|  | IMPHY004038 | −9.5 | 6.97 | 0.4524 |
|  | IMPHY010048 | −9.4 | 6.89 | 0.4273 |
|  | IMPHY013049 | −9.4 | 6.89 | 0.4273 |
|  | IMPHY001869 | −9.4 | 6.89 | 0.4947 |
|  | IMPHY004939 | −9.4 | 6.89 | 0.47 |
|  | IMPHY005185 | −9.4 | 6.89 | 0.2848 |
|  | IMPHY006963 | −9.4 | 6.89 | 0.47 |
|  | IMPHY014179 | −9.4 | 6.89 | 0.4273 |
|  | IMPHY000265 | −9.3 | 6.82 | 0.4429 |
|  | IMPHY000342 | −9.3 | 6.82 | 0.3577 |
|  | IMPHY010592 | −9.3 | 6.82 | 0.4895 |
|  | IMPHY007483 | −9.3 | 6.82 | 0.465 |
|  | IMPHY004871 | −9.3 | 6.82 | 0.4429 |
|  | IMPHY008743 | −9.3 | 6.82 | 0.4227 |
|  | IMPHY000565 | −9.2 | 6.75 | 0.46 |
|  | IMPHY015759 | −9.2 | 6.75 | 0.4182 |
|  | IMPHY012406 | −9.2 | 6.75 | 0.4182 |
|  | IMPHY000782 | −9.2 | 6.75 | 0.46 |
|  | IMPHY003706 | −9.2 | 6.75 | 0.2556 |
|  | IMPHY007248 | −9.2 | 6.75 | 0.5111 |
|  | IMPHY011489 | −9.2 | 6.75 | 0.5111 |
|  | IMPHY000908 | −9.2 | 6.75 | 0.46 |
|  | IMPHY003078 | −9.2 | 6.75 | 0.2968 |
|  | IMPHY003444 | −9.2 | 6.75 | 0.3833 |
|  | IMPHY001085 | −9.2 | 6.75 | 0.3833 |
|  | **IMPHY000366** | **−9.1** | **6.67** | **0.2935** |
|  | IMPHY008900 | −9.1 | 6.67 | 0.2676 |
|  | IMPHY003213 | −9.1 | 6.67 | 0.325 |
|  | IMPHY008619 | −9.1 | 6.67 | 0.26 |
|  | IMPHY011993 | -9.1 | 6.67 | 0.35 |
|  | IMPHY000529 | -9.1 | 6.67 | 0.364 |
|  | IMPHY000529 | -9.1 | 6.67 | 0.364 |
|  | IMPHY001837 | -9.1 | 6.67 | 0.455 |
|  | IMPHY002002 | -9.1 | 6.67 | 0.455 |
|  | IMPHY006225 | -9.1 | 6.67 | 0.35 |
|  | IMPHY009071 | -9.1 | 6.67 | 0.35 |
|  | IMPHY013909 | -9.1 | 6.67 | 0.4333 |
|  | IMPHY005067 | -9.1 | 6.67 | 0.4333 |
|  | IMPHY000053 | -9.1 | 6.67 | 0.4136 |
|  | VX-680 | −8.3 | 6.09 | 0.2515 |

**Table S2**: Selected hits and a control molecule with their docking scores with CDK1

| **S. No.** | **Compound Name** | **Affinity (kcal/mol)** | **pKi** | **Ligand Efficiency (kcal/mol/non-H atom)** |
| --- | --- | --- | --- | --- |
|  | IMPHY010379 | −12.6 | 9.24 | 0.42 |
|  | IMPHY000353 | −11.3 | 8.29 | 0.3767 |
|  | IMPHY012210 | −10.9 | 7.99 | 0.4542 |
|  | IMPHY006882 | −10.8 | 7.92 | 0.3857 |
|  | IMPHY010369 | −10.8 | 7.92 | 0.36 |
|  | IMPHY011318 | −10.8 | 7.92 | 0.4154 |
|  | IMPHY011344 | −10.8 | 7.92 | 0.3484 |
|  | IMPHY007900 | −10.8 | 7.92 | 0.3484 |
|  | IMPHY013251 | −10.7 | 7.85 | 0.4864 |
|  | **IMPHY000366** | **−10.7** | **7.85** | **0.3452** |
|  | IMPHY009264 | −10.7 | 7.85 | 0.3344 |
|  | IMPHY010476 | −10.7 | 7.85 | 0.3242 |
|  | IMPHY003878 | −10.7 | 7.85 | 0.3821 |
|  | IMPHY006541 | −10.7 | 7.85 | 0.3567 |
|  | IMPHY009261 | −10.7 | 7.85 | 0.4458 |
|  | IMPHY001660 | −10.7 | 7.85 | 0.428 |
|  | IMPHY007737 | −10.7 | 7.85 | 0.369 |
|  | IMPHY010936 | −10.7 | 7.85 | 0.369 |
|  | IMPHY007734 | −10.7 | 7.85 | 0.3567 |
|  | IMPHY005976 | −10.6 | 7.77 | 0.3655 |
|  | IMPHY007002 | −10.6 | 7.77 | 0.4077 |
|  | IMPHY001734 | −10.6 | 7.77 | 0.3419 |
|  | IMPHY005221 | −10.6 | 7.77 | 0.3926 |
|  | IMPHY005229 | −10.6 | 7.77 | 0.4077 |
|  | IMPHY005775 | −10.6 | 7.77 | 0.3419 |
|  | IMPHY007336 | −10.6 | 7.77 | 0.4417 |
|  | IMPHY002305 | −10.6 | 7.77 | 0.3029 |
|  | IMPHY010395 | −10.6 | 7.77 | 0.3419 |
|  | IMPHY011458 | −10.6 | 7.77 | 0.4077 |
|  | IMPHY009438 | −10.6 | 7.77 | 0.3212 |
|  | IMPHY000403 | −10.6 | 7.77 | 0.3533 |
|  | IMPHY002300 | −10.5 | 7.7 | 0.3889 |
|  | IMPHY005478 | −10.5 | 7.7 | 0.42 |
|  | IMPHY001575 | −10.5 | 7.7 | 0.3387 |
|  | IMPHY011162 | −10.5 | 7.7 | 0.3387 |
|  | IMPHY002477 | −10.5 | 7.7 | 0.3182 |
|  | IMPHY002700 | −10.5 | 7.7 | 0.3088 |
|  | IMPHY004082 | −10.5 | 7.7 | 0.3387 |
|  | IMPHY004287 | −10.5 | 7.7 | 0.3 |
|  | IMPHY005785 | −10.5 | 7.7 | 0.3387 |
|  | IMPHY011402 | -10.5 | 7.7 | 0.3281 |
|  | IMPHY014734 | -10.5 | 7.7 | 0.3387 |
|  | IMPHY011298 | -10.5 | 7.7 | 0.3621 |
|  | IMPHY011942 | -10.5 | 7.7 | 0.3281 |
|  | IMPHY013094 | -10.4 | 7.63 | 0.4333 |
|  | IMPHY002449 | -10.4 | 7.63 | 0.3714 |
|  | IMPHY002966 | -10.4 | 7.63 | 0.3852 |
|  | IMPHY005503 | -10.4 | 7.63 | 0.4 |
|  | IMPHY008030 | -10.4 | 7.63 | 0.3355 |
|  | IMPHY010721 | -10.4 | 7.63 | 0.416 |
|  | RO-3306 | −9.2 | 6.75 | 0.3833 |

**Table S3.** Repeated docking of Jervine with AURKB using different random seed values to assess reproducibility of binding scores.

| **S.No.** | **Random Seed** | **Affinity (kcal/mol)** | **pKi** | **Ligand Efficiency (kcal/mol/non-H atom)** |
| --- | --- | --- | --- | --- |
|  | -219310400 | -9.1 | 6.67 | 0.2935 |
|  | 645421552 | -9.1 | 6.67 | 0.2935 |
|  | -1483443896 | -9.1 | 6.67 | 0.2935 |
|  | -1492341504 | -9.1 | 6.67 | 0.2935 |
|  | -1759177624 | -9.1 | 6.67 | 0.2935 |

**Table S4.** Repeated docking of Jervine with CDK1 using different random seed values to assess reproducibility of binding scores.

| **S.No.** | **Random Seed** | **Affinity (kcal/mol)** | **pKi** | **Ligand Efficiency (kcal/mol/non-H atom)** |
| --- | --- | --- | --- | --- |
|  | 283857840 | -10.7 | 7.85 | 0.3452 |
|  | -1777147260 | -10.7 | 7.85 | 0.3452 |
|  | -1599569984 | -10.7 | 7.85 | 0.3452 |
|  | 1875290144 | -10.7 | 7.85 | 0.3452 |
|  | -866132376 | -10.7 | 7.85 | 0.3452 |

**Table S5:** Physicochemical and pharmacokinetic properties of Jervine

| **S. No.** | **Physicochemical Parameters** | **Value** |
| --- | --- | --- |
| 1. | Molecular formula | C27H39NO3 |
| 2. | Molecular weight | 425.60 g/mol |
| 3. | Number of heavy atoms | 31 |
| 4. | Rotatable bonds | 0 |
| 5. | Hydrogen acceptor | 4 |
| 6. | Hydrogen donor | 2 |
| 7. | Molar refractivity | 127.21 |
| 8. | TPSA | 58.56 Å² |
| **Lipophilicity** | | |
| 1 | iLOGP*_o/w_ (iLOGP)* | 3.89 |
| 2. | iLOGP*_o/w_ (XLOGP3)* | 2.91 |
| 3. | iLOGP*_o/w_ (WLOGP)* | 3.80 |
| 4. | iLOGP*_o/w_ (MLOGP)* | 3.48 |
| 5. | iLOGP*_o/w_ (SILICOS-IT)* | 3.65 |
| **Drug likeliness and Medicinal Chemistry** | | |
| 1. | Lipinski violations | 0 |
| 2. | PAINS | 0 |
| 3. | Ghose | Yes |
| 4. | Bioavailability score | 0.55 |
| 5. | Volume of Distribution (logL/kg) | 0.43 |
| 6. | Synthetic accessibility | 4.31 |
| 7. | Leadlikeness | No; 1 violation: MW>350 |

**Table S6.** Replica simulation of AURKB and CDK1 before and after binding with ligands.

| **System** | **RMSD (nm)** | **RMSF**  **(nm)** | ***R*g**  **(nm)** | **SASA**  **(nm^2^)** | **#Intra H-Bond** |
| --- | --- | --- | --- | --- | --- |
| AURKB | 0.420673 | 0.179939 | 2.04091 | 154.393 | 168 |
| AURKB-Jervine | 0.514653 | 0.177047 | 1.98932 | 149.977 | 173 |
| AURKB-VX-680 | 0.427991 | 0.210091 | 1.98774 | 156.154 | 164 |
| CDK1 | 0.269527 | 0.128791 | 2.04114 | 154.819 | 188 |
| CDK1-Jervine | 0.187064 | 0.112601 | 2.03541 | 153.388 | 191 |
| CDK1-RO-3306 | 0.229178 | 0.100556 | 2.05175 | 153.286 | 191 |

**Table S7:** Secondary structure elements

|  | **Native AURKB** | **AURKB-Jervine** | **AURKB-VX-680** | **Native**  **CDK1** | **CDK1-Jervine** | **CDK1-RO-3306** |
| --- | --- | --- | --- | --- | --- | --- |
| Coil | 64 | 57 | 59 | 69 | 70 | 69 |
| β-sheet | 37 | 40 | 35 | 42 | 41 | 42 |
| β-bridge | 2 | 3 | 2 | 2 | 4 | 3 |
| Bend | 29 | 29 | 31 | 36 | 36 | 33 |
| Turn | 27 | 29 | 28 | 37 | 34 | 40 |
| α-helix | 87 | 84 | 87 | 87 | 86 | 84 |
| π -helix | 0 | 0 | 0 | 0 | 0 | 0 |
| 310-helix | 4 | 4 | 5 | 5 | 5 | 6 |
| PPII-Helix | 0 | 4 | 3 | 5 | 7 | 6 |


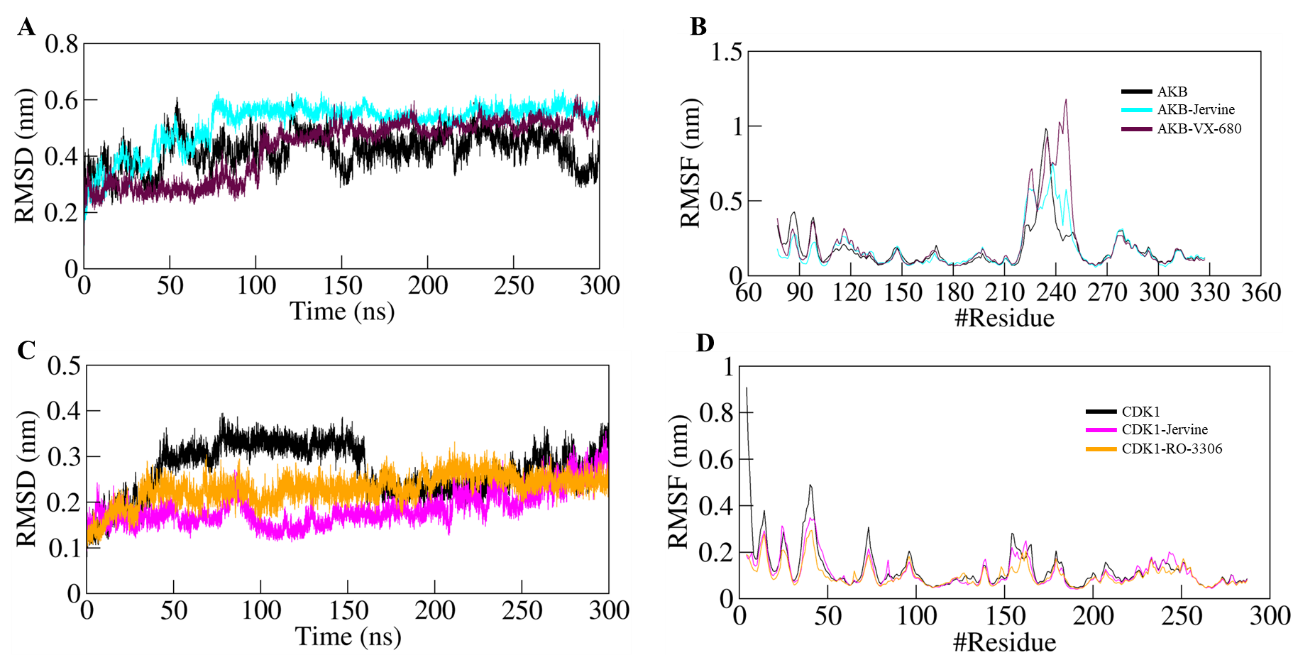


**Figure S1: (A)** RMSD plot free AURKB, AURKB-Jervine and AURKB-VX-680, **(B)** RMSF plot free AURKB, AURKB-Jervine and AURKB-VX-680, **(C)** RMSD plot of free CDK1, CDK1-Jervine and CDK1-RO-3306, **(D)** RMSF plot free CDK1, CDK1-Jervine and CDK1-RO-3306.


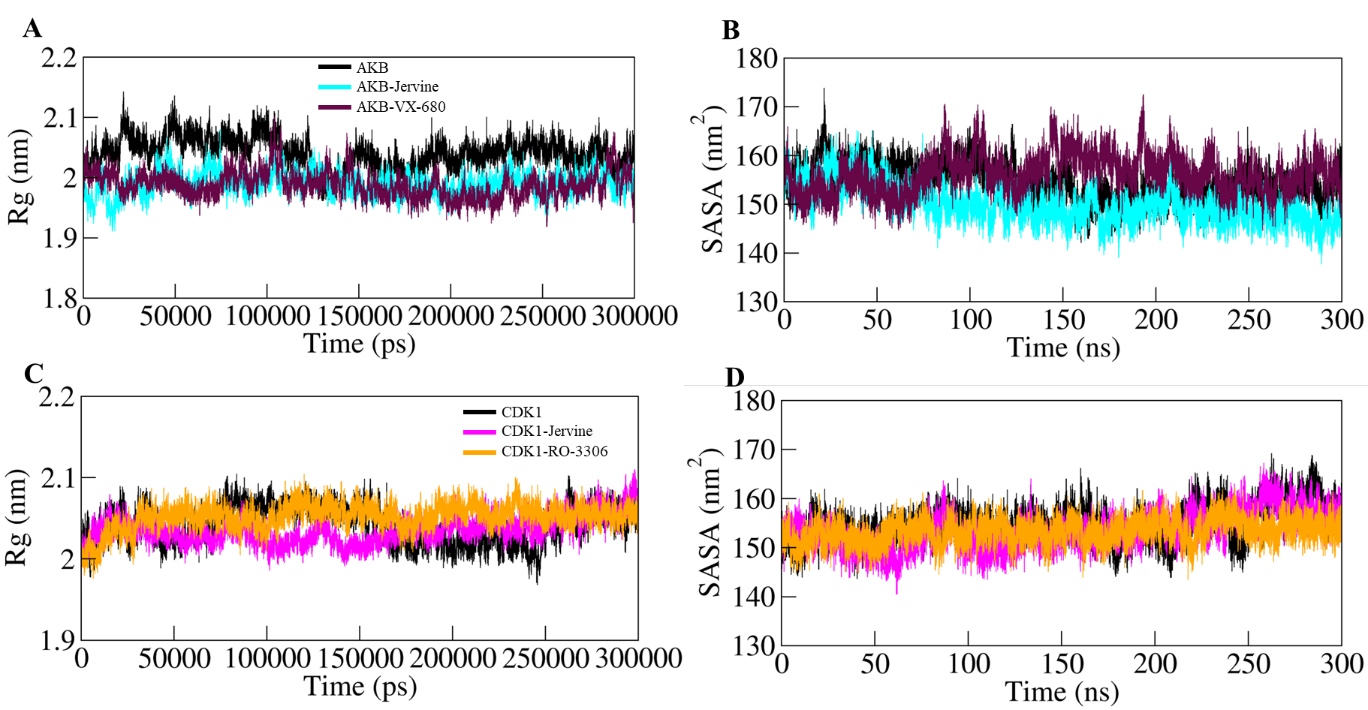


**Figure S2: (A)** *R*g plot free AURKB, AURKB-Jervine and AURKB-VX-680, **(B)** SASA plot free AURKB, AURKB-Jervine and AURKB-VX-680, **(C)** *R*g plot of free CDK1, CDK1-Jervine and CDK1-RO-3306, **(D)** SASA plot of free CDK1, CDK1-Jervine and CDK1-RO-3306


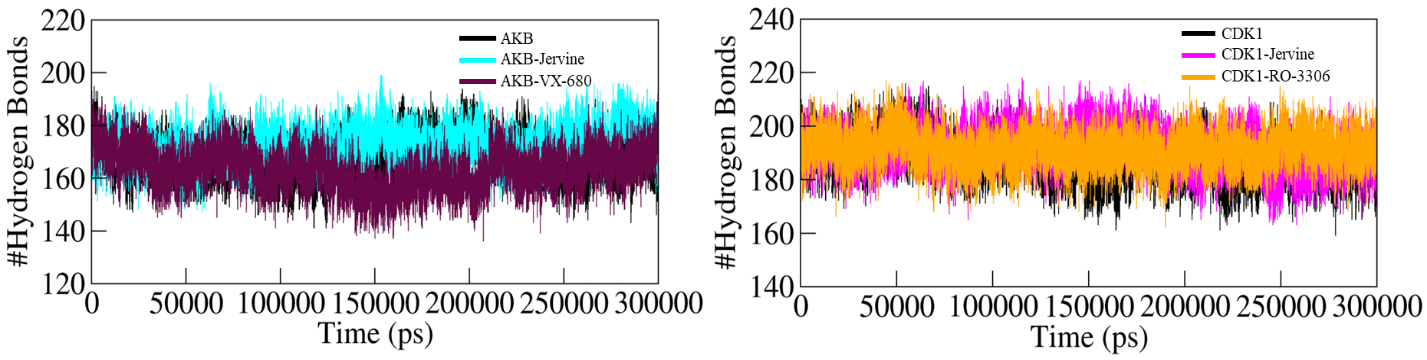


**Figure S3:** Intra-molecular hydrogen bonds **(A)** free-AURKB, AURKB-Jervine and AURKB-VX-680, **(B)** free CDK1, CDK1-Jervine and CDK1-RO-3306.


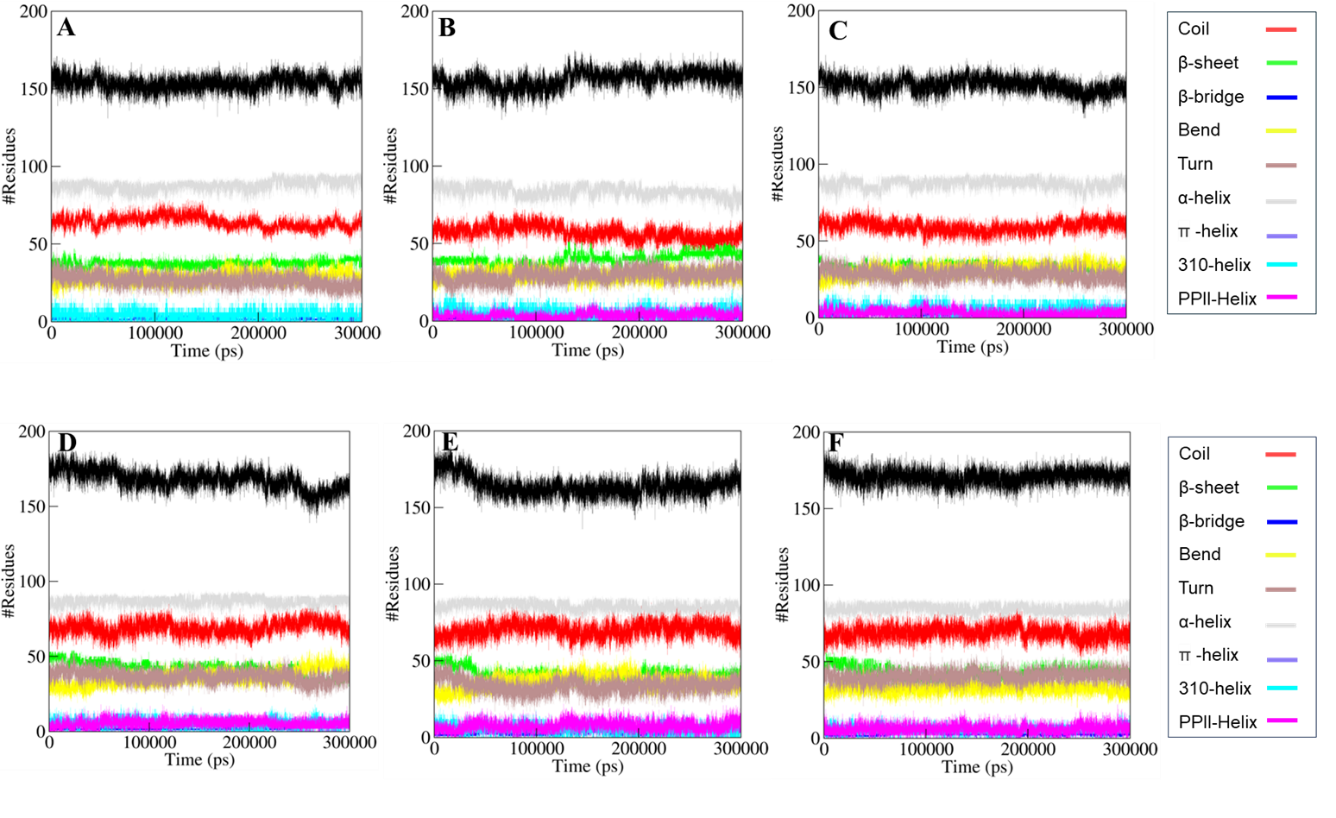


**Figure S4:** Secondary structure content of (A) free-AURKB, (B) AURKB-Jervine, (C) AURKB-VX-680, (D) free-CDK1, (E) CDK1-Jervine, (F) CDK1-RO-3306.


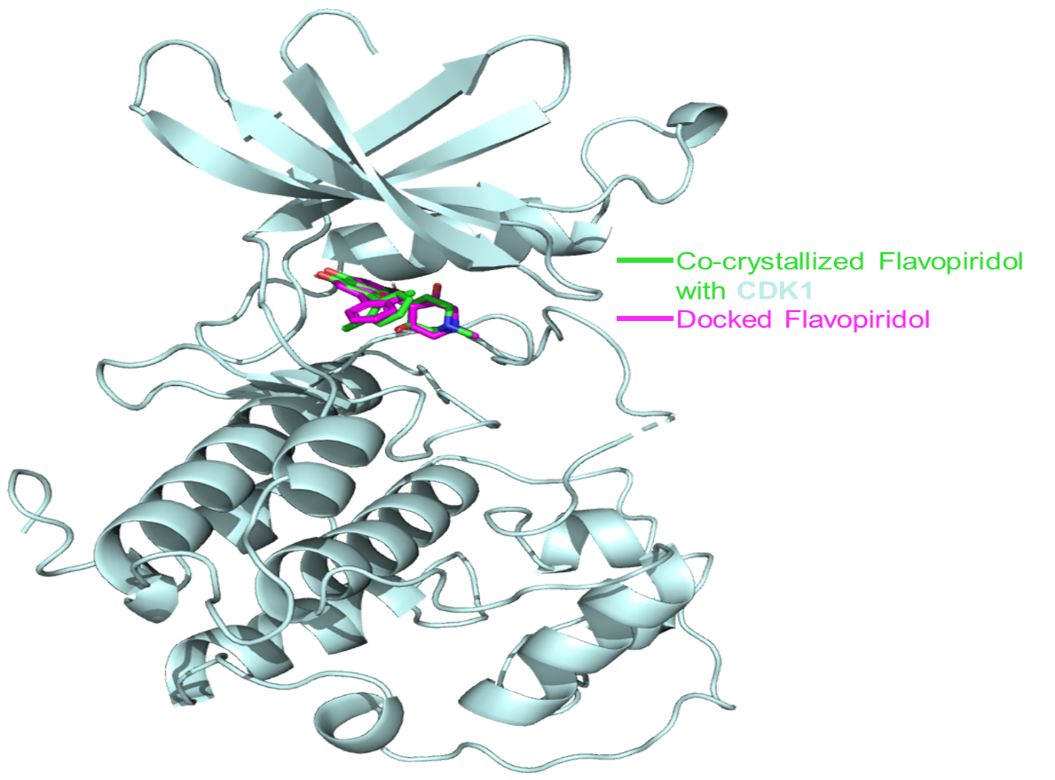


**Figure S5**
